# Functional adhesive hydrogels for biological interfaces

**DOI:** 10.1002/SMMD.20230024

**Published:** 2023-10-07

**Authors:** Changyi Liu, Kexin Peng, Yilun Wu, Fanfan Fu

**Affiliations:** ^1^ School of Environmental and Biological Engineering Nanjing University of Science and Technology Nanjing China; ^2^ College of Biotechnology and Pharmaceutical Engineering Nanjing Tech University Nanjing China; ^3^ School of Materials Science and Engineering Nanyang Technological University Singapore Singapore

**Keywords:** biological interfaces, biological sensors, hydrogel electronics, soft materials, tissue scaffolds

## Abstract

Hydrogel adhesives are extensively employed in biological interfaces such as epidermal flexible electronics, tissue engineering, and implanted device. The development of functional hydrogel adhesives is a critical, yet challenging task since combining two or more attributes that seem incompatible into one adhesive hydrogel without sacrificing the hydrogel's pristine capabilities. In this Review, we highlight current developments in the fabrication of functional adhesive hydrogels, which are suitable for a variety of application scenarios, particularly those that occur underwater or on tissue/organ surface conditions. The design strategies for a multifunctional adhesive hydrogel with desirable properties including underwater adhesion, self‐healing, good biocompatibility, electrical conductivity, and anti‐swelling are discussed comprehensively. We then discuss the challenges faced by adhesive hydrogels, as well as their potential applications in biological interfaces. Adhesive hydrogels are the star building blocks of bio‐interface materials for individualized healthcare and other bioengineering areas.

## Graphical abstract

Adhesive hydrogels have shown great potential in biological interfaces such as epidermal flexible electronics, tissue engineering, and implanted device. In this Review, we aim to summarize the current progress of functional adhesive hydrogels and to provoke some critical thinking for addressing the challenges faced in biological interfaces.

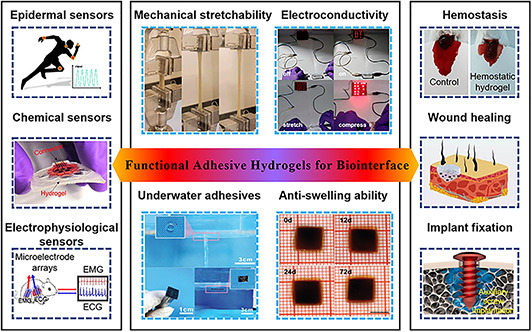

**Key points**

- The preparation methods and challenges of multi‐functional adhesive hydrogels are introduced.
- The applications of adhesive hydrogels with various functions in biological interfaces are presented.
- Challenges and future directions for adhesive hydrogel applications in bio interfaces are discussed.

## 1 INTRODUCTION

Biological interface materials, which combine bioelectronic devices with biological tissues/cells to facilitate functional exchange, have attracted extensive scientific attention in the fields of clinical medicine, advanced diagnostics, and biological research.^1^, ^2^, ^3^, ^4^, ^5^ Such biological interface materials need to be seamlessly integrated into the surfaces of tissues/cells to offer controllable mechanical compliance and efficient physical and chemical information exchange.^6^, ^7^, ^8^, ^9^ Due to their stable mechanical toughness and electrical activity, inorganic‐based materials, such as silicon and metals, are still the mainstream building blocks for bio‐interface applications.^10^, ^11^, ^12^ However, these inorganic materials encounter some serious issues, such as nonconforming contact and in vivo inflammatory responses due to their poor soft mechanical coupling and dissimilar chemical composition with biological tissues.^13^, ^14^, ^15^ It is still a huge challenge to overcome these biological barriers by only relying on inorganic materials.

Hydrogel adhesives have shown tremendous potential as biological interface materials because they naturally share traits with extracellular matrix (ECM).^16^, ^17^ The main building blocks of hydrogels are chemically or physically cross‐linked polymer chains. Generally, these polymer chains possess side chains with numerous functional groups, providing convenience for chemical modification and various interactions between chains. Therefore, hydrogels exhibit a variety of fascinating characteristics for application in bio‐interfaces, such as high‐water content, desirable elasticity, and excellent biocompatibility.^18^ For example, the high‐water content matches the features of biological tissue, dramatically boosting the interface activity and facilitating the spontaneous fusion of the material and tissue/organs.^19^ In terms of mechanical properties, the elastic modulus of the hydrogels can be controlled and adjusted in the range of 0.1–100 KPa, overlapping with most tissues and helping to eliminate mechanical mismatches between hard materials and tissue interfaces.^20^ Additionally, considering the importance of the biological activity, hydrogel adhesives can be fabricated by natural polymers with negligible immunogenicity and minor side effects, such as gelatin, silk fibroin (SF), chitin, and agarose.^21^

Numerous scientific interests have been sparked by those adhesive hydrogels with high adhesion strengths on various biological surfaces, especially under wet conditions.^22^, ^23^ Effective and strong adhesion has been constructed using a variety of techniques, including chemical, physical, and even biomimetic ones.^24^, ^25^, ^26^ However, the high moistures of hydrogels and biological surfaces make it difficult to achieve adhesion at the biological interfaces, as the existence of the interfacial fluids may separate the two surfaces at the molecular level and prevent their contact.^27^ The fundamental methods for constructing hydrogel adhesives on both dry and wet surfaces should be thoroughly investigated by utilizing/controlling the interfacial water. Meanwhile, in vivo applications are the most urgent demands for future hydrogel adhesives. In addition to having strong adhesion, the hydrogel should be hemostatic, limit interstitial fluid leakage, and prevent interface exchanges between infectious or contaminated substances and the environment around the tissue. Therefore, the development of adhesive hydrogels remains an important and challenging task. This often requires the combination of specious mutually exclusive properties in one hydrogel, such as strong adhesion and the adaptation to tissue/organ remodeling process.

Apart from the high adhesion strengths, hydrogel adhesives need to perform some other functions to meet the demands of interface applications.^28^ For instance, when using hydrogel adhesive as a wound dressing, it must have good biocompatibility (non‐allergic and non‐toxic).^29^, ^30^ In this scenario, it also needs to have functions such as antibacterial, anti‐oxidation, thermal insulation, and comfortable softness. An ideal hydrogel adhesive for wound dressings may also need to be reusable, cleanable, and simple to remove without causing further harm.^31^ Adhesive hydrogels must also possess other functionalities such as self‐healing, electrical conductivity, anti‐swelling, and stimulus responsiveness in order to be employed effectively in applications such as bone regeneration, nerve stimulation, controllable drug release, and other bio‐interface applications.^32^, ^33^, ^34^, ^35^

**FIGURE 1:**
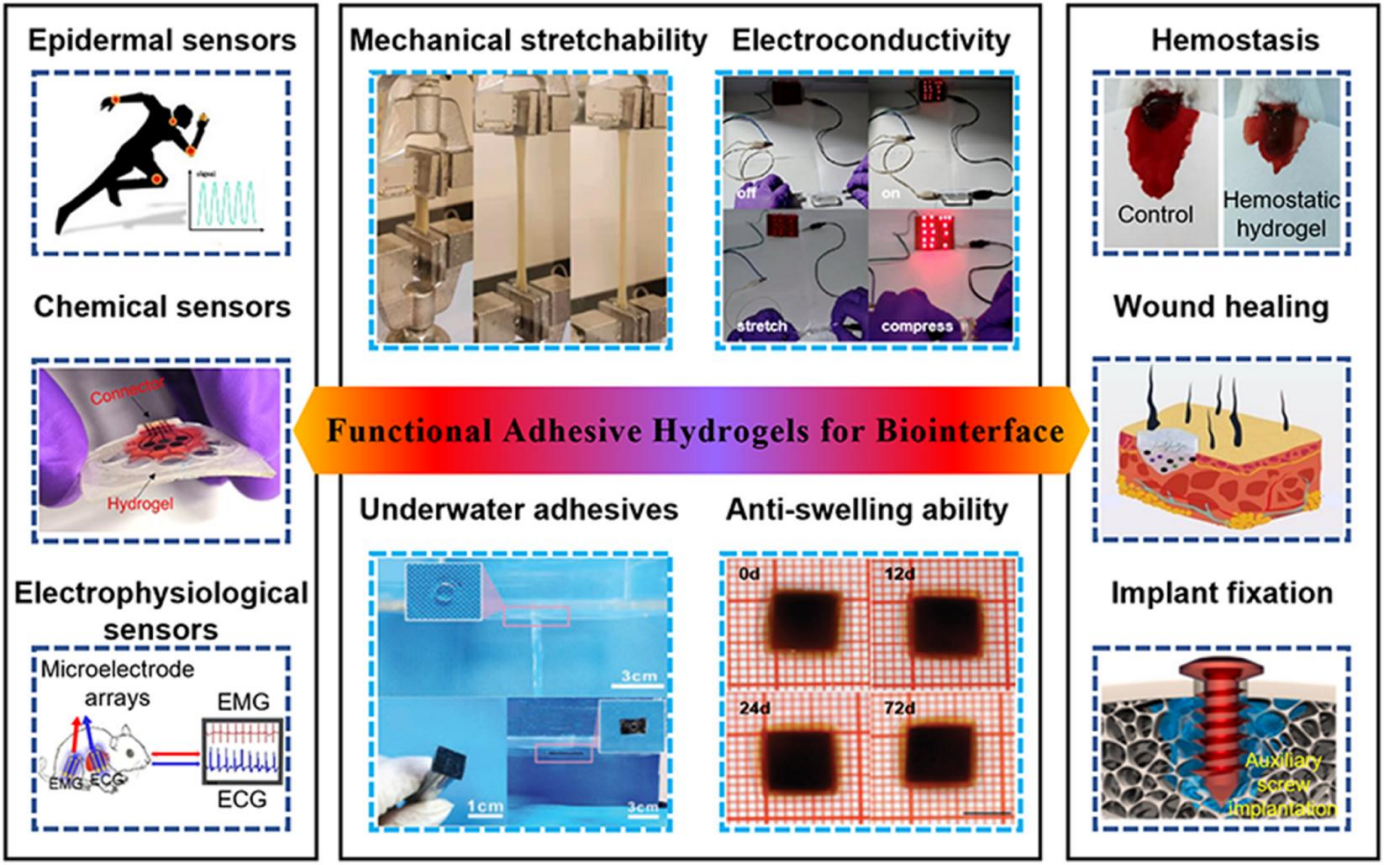
Functional adhesive hydrogels for biological interface applications. The left column displays the adhesive hydrogels used in vivo/vitro signal monitoring (epidermal sensors,^36^ chemical sensors,^37^ and electrophysiological sensors,^38^ respectively). Reproduced with permission from Ref. 36. Copyright 2023, American Chemical Society. Reproduced with permission from Ref. 37. Copyright 2022, American Chemical Society. Reproduced with permission from Ref. 38. Copyright 2022, American Chemical Society. The middle column lists the representative functions of adhesive hydrogels in practical application (mechanical stretchability,^39^ electro‐conductivity,^40^ underwater adhesives,^41^ and anti‐swelling ability^25^). Reproduced with permission from Ref. 39. Copyright 2022, Elsevier. Reproduced with permission from Ref. 40. Copyright 2022, American Chemical Society. Reproduced with permission from Ref. 41. Copyright 2022, Wiley‐VCH. Reproduced with permission from Ref. 25. Copyright 2022, Wiley‐VCH. The left column displays the adhesive hydrogels used in tissue engineering (tissue hemostasis,^42^ wound healing,^43^ and scaffolds for orthopedic implants^44^). Reproduced with permission from Ref. 42. Copyright 2022, Elsevier. Reproduced with permission from Ref. 43. Copyright 2023, American Chemical Society. Reproduced with permission from Ref. 44. Copyright 2022, The Authors, published by Elsevier.

In this review, we provide an overview of recent advances in multifunctional adhesive hydrogels for biointerfaces, with a focus on bioelectronics and tissue engineering applications (Figure 1). We emphasize the strategies for preparing functional adhesive hydrogels by using chemical, physical, and biomimetic processes to satisfy the requirements of different biological interfaces without compromising their other properties. This Review aims to provide guidelines and insights for the design of multifunctional adhesive hydrogels for various biomedical applications.

## 2 MECHANISM OF ADHESIVE HYDROGELS

**FIGURE 2:**
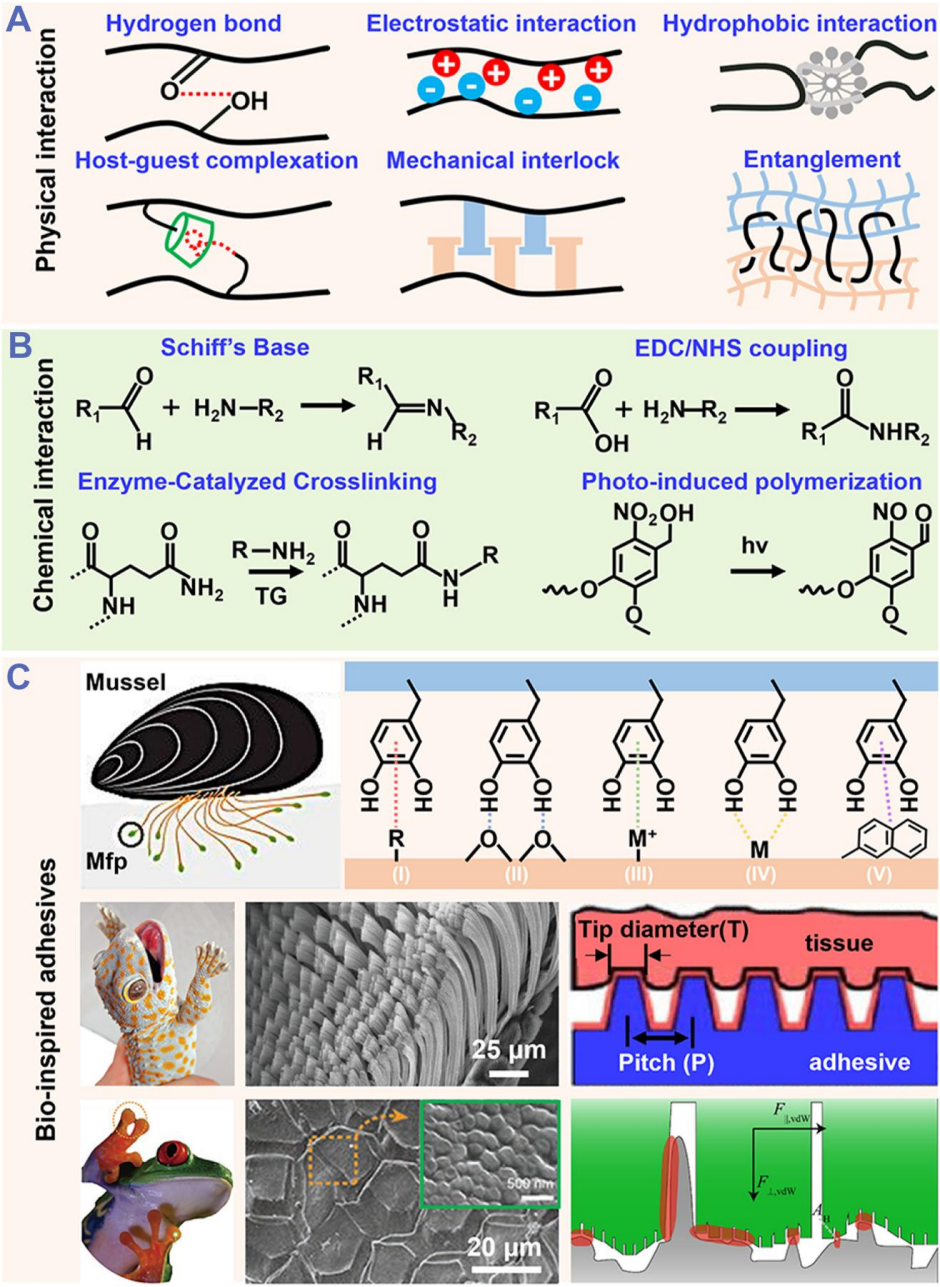
The adhesive mechanism and constructing strategies of adhesive hydrogels for biological interfaces. (A) Physical interaction: adhesive hydrogel prepared by forming mechanical interlocks or molecular interactions, such as hydrogen bound, hydrophobic/electrostatic interactions, and entanglement interlocks. (B) Chemical interaction: adhesive hydrogel prepared by forming a chemical bond based on reactions such as Schiff's Base reaction, N‐ethyl‐N’ ‐(3‐(dimethylamino)propyl)carbodiimide/N‐hydroxysuccinimide (EDC/NHS) coupling, enzyme‐catalyzed crosslinking, and polymerization. (C) Bio‐inspired adhesives: adhesive hydrogels derived from mussel,^45^ gecko,^46^, ^47^ and tree frog.^48^, ^49^ Reproduced with permission from Ref. 45. Copyright 2014, Springer Nature. Reproduced with permission from Ref. 46. Copyright 2012, Wiley‐VCH. Reproduced with permission from Ref. 47. Copyright 2008, The National Academy of Sciences of the USA. Reproduced with permission from Ref. 48. Copyright 2019, Wiley‐VCH. Reproduced with permission from Ref. 49. Copyright 2018, The Authors, published by Springer Nature.

Adhesive hydrogels are usually achieved by establishing strong physical interaction or chemical bonds between the hydrogel polymers and the targeted substrates, as well as the unique topological structures inspired by nature (Figure 2). Considering the distinction between dry and wet interfaces, different adhesive hydrogels have different design approaches and priorities. In general, the main mechanisms for those physical interaction‐based adhesive hydrogels are the hydrogen bond,^50^, ^51^ hydrophobic/electrostatic interactions,^24^, ^52^ host‐guest complexation,^53^ and the entanglement interlock from polymer network/microstructure.^54^, ^55^ The diversity of physical interaction enables variable designs to fulfill instant and tough adhesion between different biological interfaces. As reported by Suo's team, a “molecular Velcro” on polydimethylsiloxane substrates was fabricated, endowing the adhesion to polyacrylamide (PAAm) hydrogels immediately with a controllable adhesion energy via hydrogen bonds.^56^ This hydrogel adhesive was reversible in response to pH or temperature changes, providing a feasible approach for the detachment designs.

Notably, physical interaction‐based adhesives can be disrupted when applied in a harsh microenvironment. Some physical interactions, such as hydrogen bonds, will be weakened in a moist environment because the interfacial water will preferentially form hydrogen bonds with the hydrogel or the adhesive substrate. One approach to dealing with such issues is to design an adhesive hydrogel with special functionality. For example, Liu et al. developed a Janus adhesive hydrogel by using a gradient polyelectrolyte complexation approach that causes a phase separation between the hydrogel's top and bottom.^57^ The resulting adhesive hydrogel showed double‐sided properties, one of which is hydrophilic and the other is hydrophobic. The hydrophobic properties of the hydrogel surface can be exploited to keep out interfacial water or block access, which helps the functional groups (carboxyl) on the surface effectively form hydrogen bonds with tissues. On another hydrophilic side (as opposed to the hydrophobic side), the hydrogel's carboxyl groups could be highly neutralized by polycations, resulting in a loss of adhesion. In this case, this hydrophilic side can also serve as an isolating layer to prevent postoperative/inter‐organ adhesions.

Adhesive hydrogels made from both synthetic and natural polymers have been widely modified with various functional groups to form chemical bonds with targeted substrates. The chemical cross‐linking mechanisms such as Schiff's Base, free radical polymerization, N‐ethyl‐N’‐(3‐(dimethylamino)propyl)carbodiimide/N‐hydroxysuccinimide (EDC/NHS) coupling, and enzyme‐catalyzed crosslinking reactions are the common chemical reactions for building a tough adhesive.^58^, ^59^, ^60^ Depending on the reversibility of chemical bonds, those adhesive hydrogels can be classified as chemical cross‐linking and dynamic cross‐linking.^61^ Adhesive hydrogels achieved by chemical cross‐linking typically have physiological and mechanical stability. However, when developing such adhesive hydrogels, the biological activity of the chemical reaction process should be seriously considered, such as the cytotoxicity of free radical type and the non‐degradability of some chemical crosslinks, especially for their application in tissue engineering. In contrast, adhesive hydrogels achieved by dynamic crosslinks can exhibit both the physiological and mechanical stability of covalent bonds and the programmable reversibility of noncovalent bonds in a controllable environment. For example, the Schiff's base reaction is a rapid, mild, and pH‐dependent (occurs at weakly acidic pH conditions) reaction between the aldehyde/ketone group and amino group. Taking advantage of the high reactivity and selectivity, adhesive hydrogels based on Schiff's base reaction are promising in biological tissue engineering. Due to the abundant amino groups on the surface of tissues/organs, polymers with aldehyde/ketone groups, or those that can be converted into derivatives, are widely used to develop functional tissue hydrogel adhesives.^62^, ^63^ The resulting hydrogel tissue adhesive typically includes non‐invasive de‐paste functions, providing a desirable and alternative option for tissue engineering.

Additionally, nature offers countless inspirations and strategies for creating and designing adhesive hydrogels, particularly for marine organisms like mussels and sandcastle worms.^64^, ^65^ The adhesive proteins of marine mussels are rich in the amino acid L‐3,4‐dihydroxyphenylalanine (Dopa), exhibiting strong wet adhesion on various surfaces through Dopa‐mediated interfacial binding.^66^ Since the interaction mechanism of Dopa action has been extensively elucidated, these adhesive proteins have been a major source of inspiration for the development of biomimetic wet hydrogel adhesives. A significant amount of hydrogel adhesives focus on mussel‐inspired polymers.^67^, ^68^ Beyond exploring the chemistry‐based adhesives, terrestrial animals also provide intrinsic physical interaction mechanisms for multifunctional adhesives. For example, geckos achieve strong adhesion and rapid peeling ability to the substrate by using the foot with microscopic hierarchical structures that can control the van der Waals' force in motion.^47^ And, thanks to the hexagonal pattern pillars in toe pads, tree frog possesses increased friction against wet surfaces and shows strong adhesion beneath moving liquids.^49^ Insights and understandings from the interaction mechanisms of nature examples will pave the way for the design and application of functional hydrogel adhesives.

## 3 ENGINEERING MULTIFUNCTIONAL ADHESIVE HYDROGEL

### 3.1 Biocompatible adhesive hydrogels

Adhesive hydrogels are widely used in biomedical fields such as implants, in vivo imaging and diagnostic systems, drug and gene delivery systems, and tissue engineering, where adhesive hydrogels are required to have good biocompatibility and low immune response.^21^, ^69^ Both natural and synthetic polymer‐based adhesive hydrogels seek to maximize their strengths, not only in terms of the biological activity of the polymer itself, but also in terms of cross‐linking chemical reagents, by‐products and degradation products. Adhesive hydrogels based on synthetic polymers (e.g. polylactic acid, polyethylene glycol [PEG]) can have controllable chemical/physical properties. However, the biocompatibility of these polymers is generally lower than that of natural polymers. Natural polymer hydrogels, including proteins, chitosan (CS), alginate, and others, not only come from a variety of sources and have good biocompatibility, but also help to identify the interaction between hydrogels and tissues and actively stimulate tissue regeneration.^69^

**FIGURE 3:**
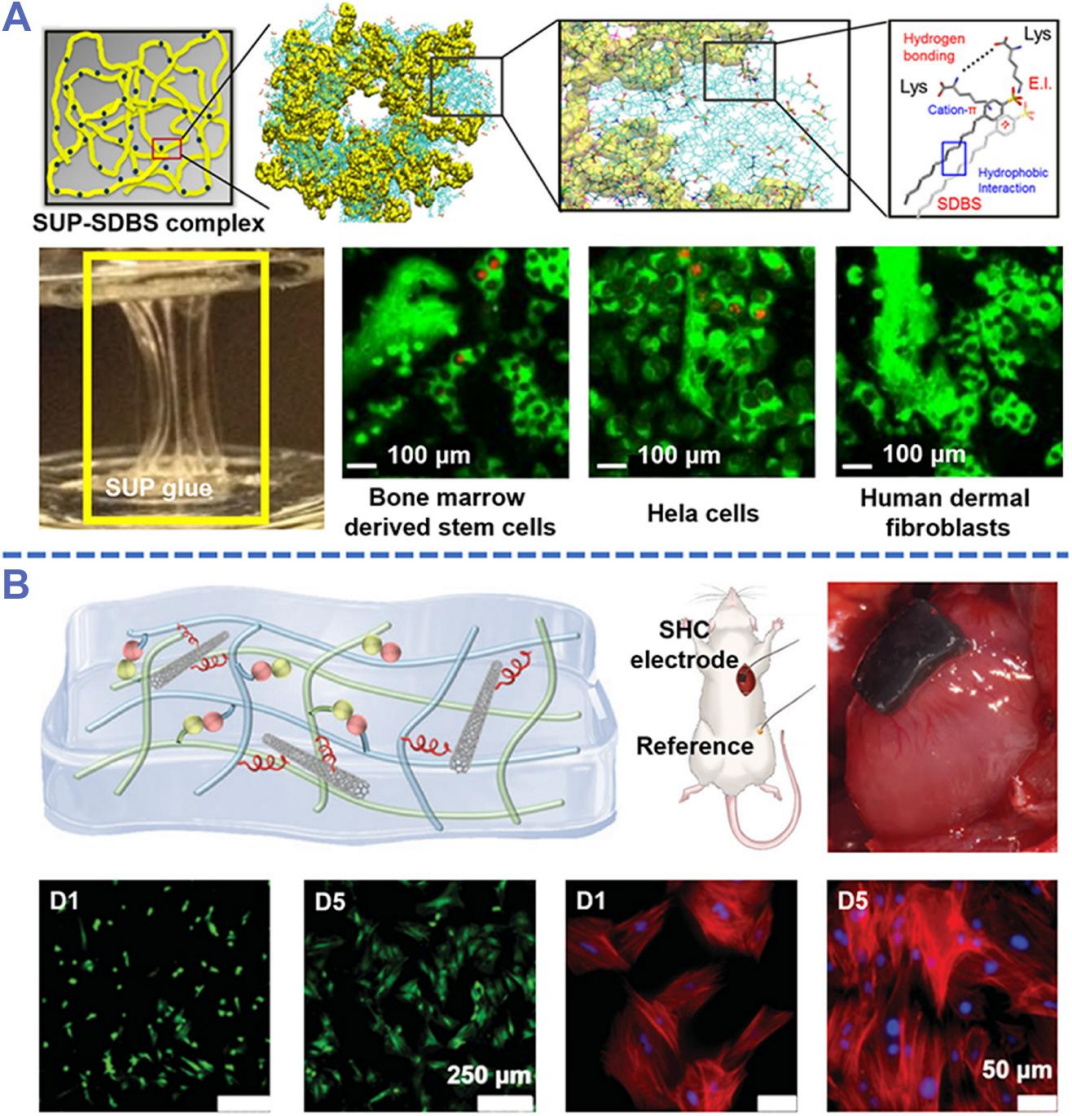
Biocompatible adhesive hydrogels. (A) A biocompatible hydrogel cross‐linked by physical interaction for cell adhesion and culture. This schematic diagram reflects the cohesion and adhesion mechanism of SUP‐SDBS (the yellow represents SUPs, and the blue represents SDBS). Reproduced with permission from Ref. 70. Copyright 2021, The Authors, published by Springer Nature. (B) Adhesive hydrogel composited with carbon nanotubes for cardiac engineering. Dynamic cross‐linking between silk fibrin and HA‐CHO leads to the formation of biocompatible adhesive hydrogels. Reproduced with permission from Ref. 71. Copyright 2022, The Royal Society of Chemistry. HA‐CHO, hyaluronic acid‐aldehyde; SDBS, sodium dodecylbenzene sulfonate; SUPs, supercharged polypeptides.

Based on the natural polymers, Ma et al. developed a biocompatible adhesive hydrogel using supercharged polypeptides (SUPs) and sodium dodecylbenzene sulfonate (SDBS) as hydrogel networks, which avoided forming covalent bonds.^70^ This SUP‐SDBS adhesive hydrogel was able to strongly bond the two pieces of glass together (Figure 3A). The adhesive strength could be adjusted from 3.0 to 16.5 MPa by changing the number of SUPs in the hydrogel. This is due to the contribution of free lysine in the SUPs to the adhesive strength of the hydrogel. To evaluate the biocompatibility, such a hydrogel was used as a scaffold for the culture of different cell types. The result showed the good biocompatibility of the SUP‐SDBS hydrogel by quantifying cell viability.

To improve the processability and mechanical properties, adhesive hydrogels based on natural polymers are usually modified with appropriate chemical functional groups.^72^ SF is a well‐known and highly bioactive polymer that is rich in modifiable functional groups.^73^, ^74^ Ding et al. developed an electrophysiological interface using SF and hyaluronic acid‐aldehyde (HA‐CHO).^71^ This hydrogel had good adhesion and reliable mechanical properties, allowing it to adhere to the heart, cerebral cortex, and sciatic nerve (Figure 3B). The signal‐to‐noise ratio (SNR) reached 37 when the hydrogel recorded the electrocardiogram (ECG). After co‐culture of hydrogel and bone marrow mesenchymal stem cells for 5 days, cell viability was significantly increased and a clear cytoskeleton was presented. Additionally, the excellent electrical conductivity and biocompatibility enable it to be used as an implantable flexible electrode for recording bioelectrical signals and stimulating nerves. The results showed that mild chemical modification or grafting of natural polymer functional groups is one of the most effective ways to achieve improved performance, and added functionality. At the same time, the biological activity of the chemical process, by‐products, and degradation products must be taken into account. This is also one of the biggest challenges for currently available biocompatible adhesive hydrogels, including both natural and synthetic‐based polymers.

### 3.2 Tough and stretchable hydrogels

The adhesiveness of hydrogels depends on the synergy between adhesion and cohesion properties.^75^, ^76^ The adhesive force is the force between the hydrogel and the substance. The cohesive force is related to the intermolecular interaction in the polymer network within the hydrogel, which is associated with the mechanical properties of the hydrogel.^33^, ^77^ Since the type and number of functional groups on the surface of the hydrogel are usually fixed, improving its mechanical properties to a certain extent will weaken its tissue adhesion.^76^, ^78^ To achieve better mechanical properties and adhesion to the substrate for the adhesive hydrogels, it is necessary to balance the relationship between adhesion and cohesion.^76^

**FIGURE 4:**
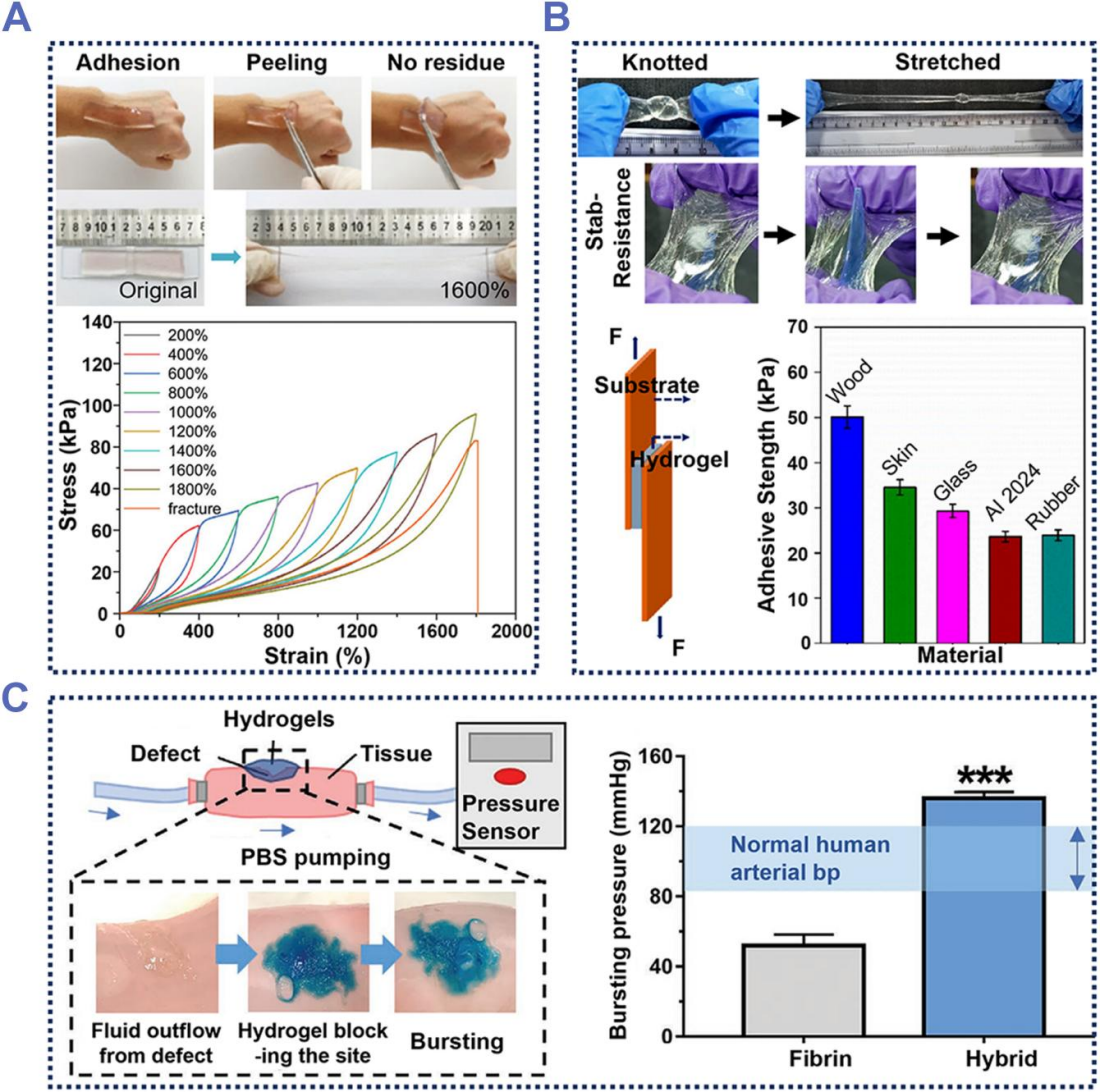
Stretchable and explosion‐proof adhesive hydrogels. (A) Adhesive hydrogel with excellent stretchability and energy dissipation mechanism. Reproduced with permission from Ref. 79. Copyright 2022, The Royal Society of Chemistry. (B) Adhesive hydrogel with excellent flexibility and stab‐resistance. Reproduced with permission from Ref. 40. Copyright 2022, American Chemical Society. (C) An adhesive hydrogel with explosion‐proof properties. The bursting pressure of this hydrogel is higher than normal human arterial blood pressure. Reproduced with permission from Ref. 80. Copyright 2022, Wiley‐VCH.

A tough hydrogel with gradient adhesion was reported by Zhang et al.,^79^ which adhered well to the skin surface (Figure 4A). This hydrogel had good mechanical properties, with a fracture stress of 93.0 KPa. It was peeled off without noticeable discomfort to the user and did not leave any residue behind. In addition, such hydrogels also had intrinsic advantages in terms of elongation, stress, and toughness. In typical continuous time loading‐unloading cycle experiments, it had been shown that hydrogels can achieve good energy dissipation and reduce the fracture of hydrogel networks.^81^ As reported by Roy et al., AAm and N‐hydroxy methyl acrylamide (HMAM) were cross‐linked with *β*‐cyclodextrin (*β*‐CD) to form the *β*‐AH hydrogel with good mechanical stability.^40^ The *β*‐AH hydrogel showed excellent adhesion. The adhesive properties of the hydrogels were measured using the lap shear test of the hydrogels (Figure 4B). The lap shear stress‐percentage displacement curves showed that this hydrogel had a good adhesion to materials such as wood, skin, and glass. Due to the dynamic and reversible hydrogen bonds between PAAm and Polymer‐HMAM (PHMAM) chains in the polymer network, this hydrogel exhibited excellent elongation, flexibility, stab resistance, and fast recovery nature. The excellent mechanical properties of *β*‐AH hydrogels may be related to the dynamic hydrogen bonds and energy dissipation mechanism formed between its polymer chains. Its energy dissipation mechanism is the breakdown of hydrophobic interactions between highly elastic hydrophobic chains. Therefore, the construction of energy dissipation mechanisms in the internal hydrogel network can significantly improve the mechanical properties of hydrogels.

Adhesive hydrogels can be used to repair blood vessels and organs.^59^, ^82^ In this case, the hydrogel must possess burst pressure resistance to withstand the pressure of the blood vessel or organ walls. Xia et al. prepared a hybrid adhesive hydrogel using HA and PEG.^80^ This adhesive hydrogel demonstrated strong adhesion to moist tissue surfaces and could withstand stretching and twisting of the tissue. Under simulated emergency bleeding conditions, the hydrogel could withstand a burst pressure of approximately 136 mmHg, which is higher than normal human arterial blood pressure (80–120 mmHg) and 260% higher than fibrin gel, which has become a commercial hemostatic product (Figure 4C). Benefiting from the good mechanical properties and rapid gelation performance, such a hydrogel had been used for rapid hemostasis in blood vessels, the digestive tract, etc. In the in vivo model evaluation of arterial bleeding, this hybrid hydrogel can effectively reduce blood loss and the risk of rebleeding. The aggregation of blood cells on the hydrogel helps to promote hemostasis. The hemostatic effect of the hydrogel can last for more than 2 days.

Anti‐fatigue property requires hydrogels to maintain adhesion, toughness and other features under cyclic mechanical loading. The fatigue resistance of anti‐fatigue hydrogels is characterized by the fatigue threshold (*G*_0_), that is, the energy release rate without fatigue cracking.^83^ To improve the anti‐fatigue properties of hydrogels, it is necessary to make the energy per unit area of hydrogel fracture much greater than the energy required to fracture single‐layer molecular chains.^84^

**FIGURE 5:**
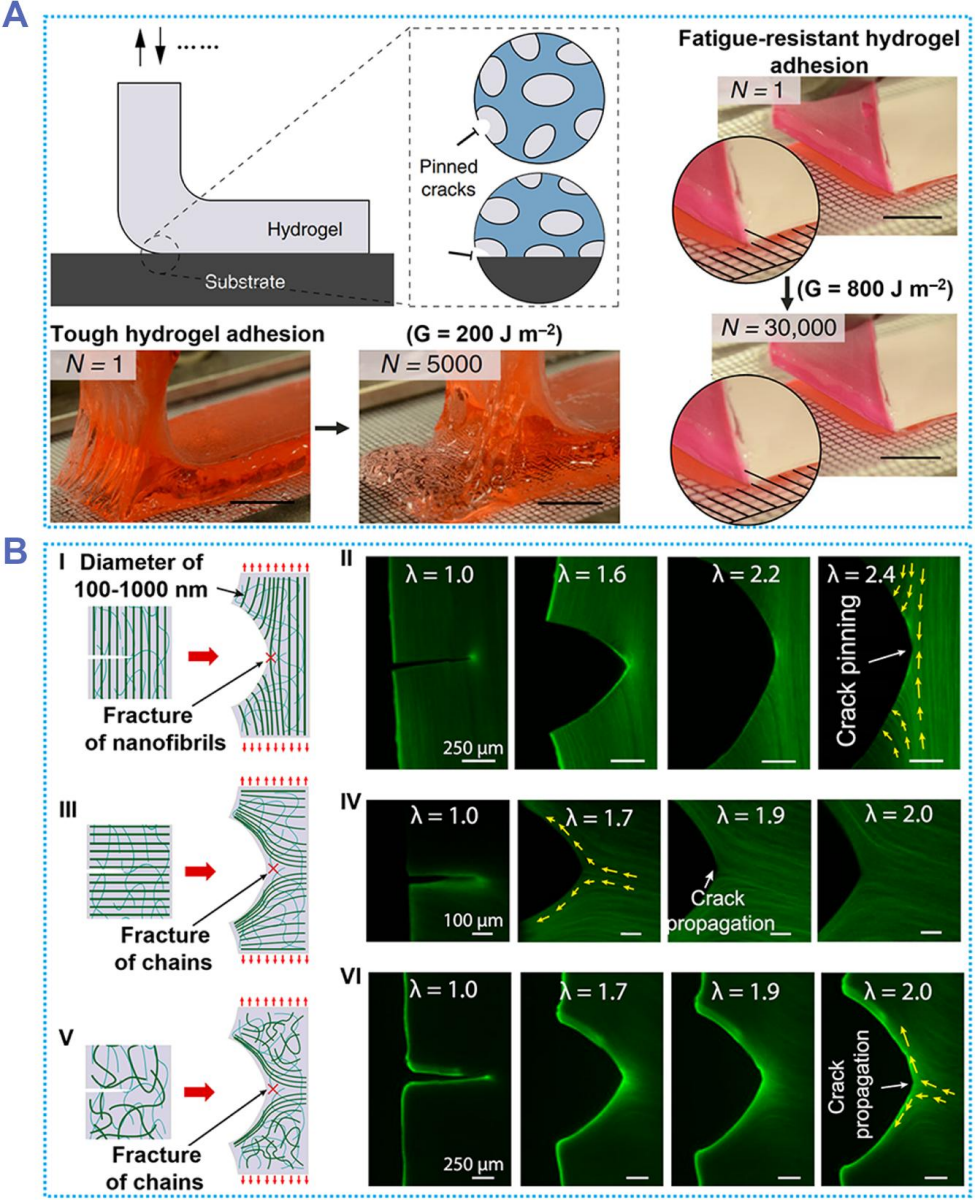
Anti‐fatigue adhesive hydrogels. (A) A fatigue‐resistant hydrogel adhesive based on nanocrystalline regions. This fatigue‐resistant hydrogel showed no fatigue expansion after 30,000 repetitions of the adhesion‐detachment experiment. Reproduced with permission from Ref. 85. Copyright 2020, The Authors, published by Springer Nature. (B) A toughness anti‐fatigue hydrogel adhesive with ordered nanofiber arrangement. When extended perpendicular to the nanofiber direction, this fatigue‐resistant hydrogel adhesive exhibited little fatigue expansion. Reproduced with permission from Ref. 86. Copyright 2019, The National Academy of Sciences of the USA.

Biological tissues such as muscles have excellent fatigue resistance properties. This may be due to the fact that the collagen fibers in the tissue are well ordered and partially crystallized.^84^, ^85^ Zhao et al. proposed that the introduction of nanocrystalline regions into hydrogels can effectively improve their anti‐fatigue adhesion.^85^ The hydrogel was immobilized on the substrate and the nanocrystalline regions formed by dry annealing could pin the hydrogel and substrate, resulting in fatigue‐resistant adhesion (Figure 5A). The *G*_0_ of the hydrogel interface for anti‐fatigue adhesion is 800 J/m^2^, which is consistent with the adhesion of the joint tissue in vivo.^87^ There was no observable crack propagation on the surface of the hydrogel after 30,000 cycles with an energy release rate of 800 J/m^2^. On the contrary, after 5000 cycles of 200 J/m^2^ energy were applied to ordinary tough hydrogels, the crack propagation had already led to the fatigue fracture. In addition, the reciprocating normal compressive load (compressive stress is about 1 MPa) was applied to the hydrogel with bone. The anti‐fatigue hydrogel had no crack propagation on the surface after 5000 cycles, while the tough hydrogel had a cohesive fracture. It is worth mentioning that this anti‐fatigue hydrogel was still stable after soaking in deionized water (DIW) for 90 days, with the original fatigue threshold.

The nanocrystalline region can also have an impact on the fatigue fracture resistance of the hydrogel.^86^ This is due to the fact that only fractured crystalline regions in the hydrogel can undergo fatigue expansion, but the energy required to fracture monolayer amorphous chains is much smaller than that required to fracture crystalline regions.^84^ Although the higher the crystallinity, the better the anti‐fatigue performance of the hydrogel, the increase in the crystal region will lead to a decrease in the water content of the hydrogel and an increase in the modulus. To address this issue, Zhao et al. proposed that pre‐stretch mechanical training could be performed on the hydrogel to induce the orderly arrangement of nanofibers, thereby achieving fatigue fracture resistance (Figure 5B).^86^ In this work, the fatigue resistance of the hydrogel is due to the ability of the nanofibers to pin cracks and prevent them from propagating. The anti‐fatigue hydrogel with the crack path perpendicular to the fiber was stretched parallel to the fiber and there was no crack propagation when the applied strain ratio (*λ*) was 2.4. In this state, the hydrogel fatigue threshold is 1250 J/m^2^, and no crack growth occurs in 30,000 tensile test cycles. However, when the hydrogel with the crack path parallel to the fiber was applied with a value of *λ* of 1.5, the crack began to propagate between the fibers. During this time, the amorphous chain between the nanofibers was broken. When the hydrogel without pre‐stretching was stretched, some randomly oriented nanofibers were gradually arranged with stretching, but these nanofibers couldn't bridge at the crack tip, and the amorphous chain broke. Additionally, this anti‐fatigue hydrogel not only achieves one direction, but also an isotropic anti‐fatigue hydrogel can be obtained by combining 3D printing technology and mechanical training. In addition to increasing their crystallinity, hydrogels can also be made more fatigue resistant by using random coiled polymers,^83^ entanglement,^10^ and the combination of crystallinity and entanglement.^88^ Anti‐fatigue hydrogels can not only be used as electrodes, implantable devices (such as prosthetics), and soft robots, but also maintain long‐term stable performance.

### 3.3 Underwater adhesive hydrogel

Adhesive hydrogels are expected to be used in tissue interface engineering, including dressings, patches, and scaffolds. For these biomedical adhesive hydrogels, robust and repeatable adhesion ability in a wet environment is critical. However, in a wet environment, particularly underwater, the water molecules on the contact surface will form hydrogen bonds with the adhesive hydrogel or the substrate preferentially, thereby inhibiting the adhesion between the hydrogel and the substrate. In addition, after a period of time in the water, the hydrogel may lose adhesion and stretchability due to water absorption. Therefore, interfacial bonding and energy dissipation are important for hydrogel adhesion in wet environments.^89^

**FIGURE 6:**
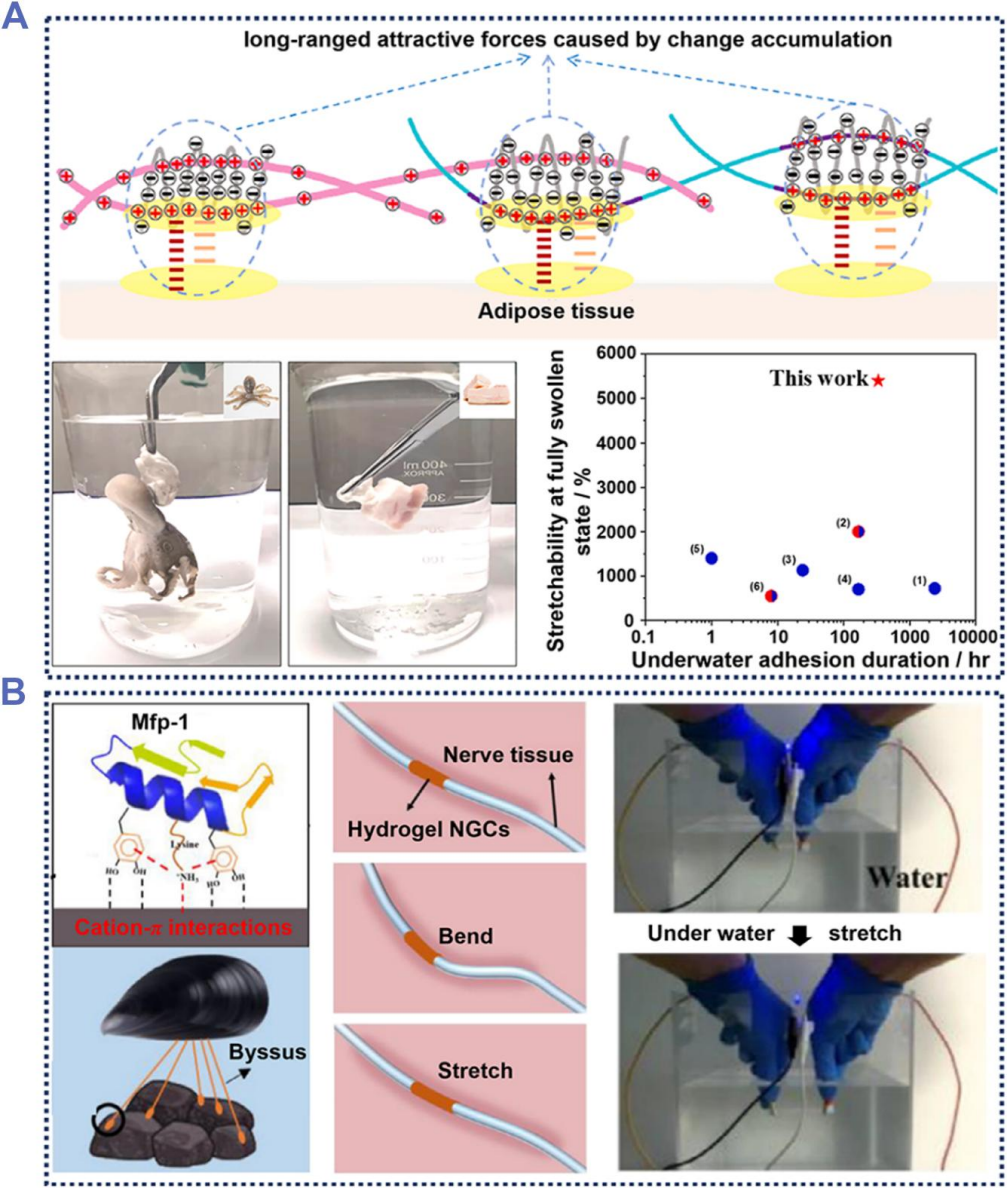
Underwater adhesive hydrogels. (A) A kind of underwater‐adhesive hydrogel based on ionic interactions. Compared with other underwater‐adhesive hydrogels, this hydrogel has excellent adhesion time and stretchability in the swollen state. Reproduced with permission from Ref. 90. Copyright 2022, Elsevier. (B) The underwater adhesive hydrogel used for nerve fiber repair. In addition to the ability to adhere underwater, the hydrogel also conducts electricity underwater. Reproduced with permission from Ref. 91. Copyright 2022, Elsevier.

On the other hand, it is also necessary to consider the specific composition of tissues and organ surfaces. For example, many underwater adhesive hydrogels have difficulty interacting with fat due to their high hydrophobicity and low density. To overcome this issue, Wang et al. introduced 2‐acryloyloxyethyltrimethyl ammonium chloride (DAC) and CS into the hydrogel to produce the adhesive hydrogel.^90^ In this work, Polymer‐DAC (PDAC) and CS in hydrogel contain positively charged primary and quaternary amine groups. These groups facilitate underwater adhesion by interacting electrostatically with the negatively charged functional groups. As a result, the hydrogel can be firmly bonded in situ to octopus, and fat in water within 30 s (Figure 6A). This underwater adhesion could be repeated more than 10 times without loss of adhesion. The adhesion of this hydrogel to fats under water may be due to electrostatic interactions, hydrophobic‐lipophilic interactions, or a synergistic effect of electrostatic and hydrophobic interactions. In addition, this hydrogel can not only remain in water for a long time, but also have good adhesion and stretchability in a fully expanded state. However, the adhesive strength of it in water containing salt (static shielding agent) was very weak. Therefore, this indicated that maintaining long‐term and stable interfacial adhesion is another serious challenge for a biological adhesive hydrogel used in a complex physiological environment such as a specific pH and salt concentration.

Inspired by the cation‐π interactions between mussel foot protein and substances in seawater, Cai et al. developed a hydrogel with excellent adhesion in seawater.^91^ Hydrogels contain positively charged groups and aromatic monomers that can form electrostatic interactions and dynamic non‐covalent bonds with substrates in an underwater environment to achieve adhesion. The underwater environment is closer to the internal environment. Varying the strain and bending of hydrogels in water can simulate how it changes in vivo. The hydrogel still conducted electricity in the water, and the brightness of the attached LED light changed when strain is applied, indicating better tensile strain sensitivity (Figure 6B). This hydrogel had been shown to have a good function in supporting nerve regeneration. In addition, there were already many wet environment adhesive hydrogels that could replace existing solutions. For example, hydrogel Janus Tough Adhesive firmly adhered to the bloody tendon and contributed to rapid wound healing,^13^ hydrogel gelatin‐polydopamine‐nano‐clay (GPC) promoted the healing of oral ulcers,^92^ double‐layered structure hydrogel was used as an underwater sensor.^93^

### 3.4 Anti‐swelling adhesive hydrogel

Adhesive hydrogels have difficulty coping with high humidity and easily swell and even degrade in aqueous environments due to the highly hydrophilic nature of hydrogel polymers. The use of adhesive hydrogels under water is strongly influenced by a swelling coefficient. The swelling of the hydrogel destroys its cross‐linked structure, increasing its volume, and significantly decreasing adhesion and mechanical properties.^24^, ^25^ Anti‐swelling is an important property required for adhesive hydrogels in biomedical applications. Otherwise, the swollen hydrogel may compress the surrounding tissue at the adhesion site, or even fall off. Therefore, the anti‐swelling properties of hydrogels can expand the application range of adhesive hydrogels.

**FIGURE 7:**
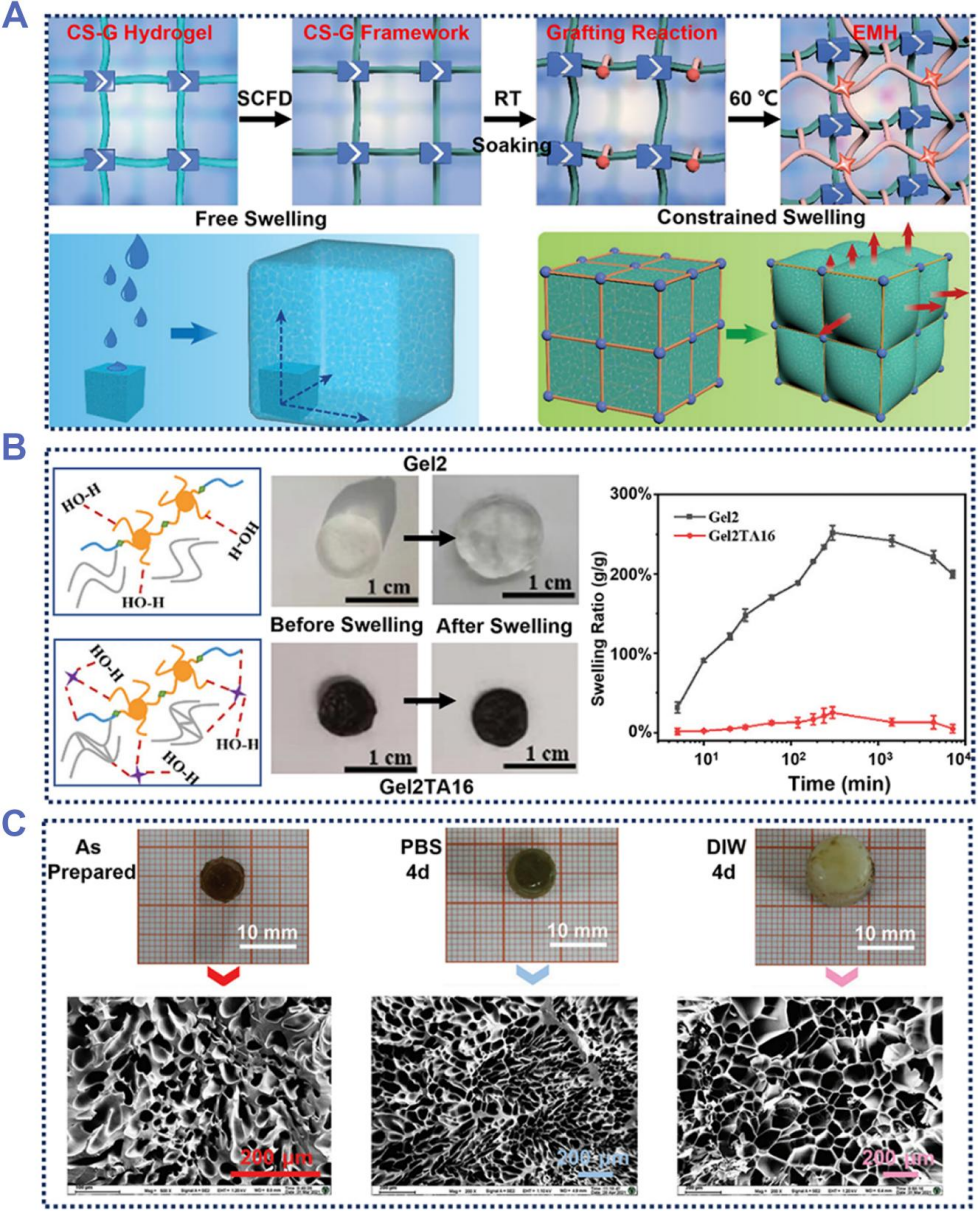
Anti‐swelling adhesive hydrogels. (A) An anti‐swelling adhesive hydrogel based on CS‐G framework. The CS‐G network outside the hydrogel can effectively prevent the free swelling of the hydrogel. Reproduced with permission from Ref. 25. Copyright 2022, Wiley‐VCH. (B) An anti‐swelling adhesive hydrogel with improved network density. Compared with the original hydrogel, the hydrogel with an increased crosslinking degree has a surprising swelling resistance. Reproduced with permission from Ref. 94. Copyright 2022, The Royal Society of Chemistry. (C) SEM images of the anti‐swelling hydrogel soaked for a period of time. Reproduced with permission from Ref. 95. Copyright 2022, The Royal Society of Chemistry.

Inspired by the ECM, which maintains low swelling through the interpenetrating structure, Wu et al. prepared a hydrogel with controllable swelling.^25^ The CS‐G network constructed from CS and Genipin had high toughness, hydrophobicity, and stability. The grafting of AAm monomers onto CS‐G resulted in a swelling resistant hydrogel. The CS‐G framework of this hydrogel firmly hooped the PAAm hydrogel, limiting its swelling (Figure 7A). As a result, almost no swelling was observed after soaking in phosphate‐buffered saline (PBS) for this hydrogel, in contrast to 500% swelling of PAAm hydrogel without CS‐G framework.

In addition to adding a frame outside the hydrogel to reduce the swelling of the hydrogel, it can also be achieved by increasing the cohesion of the hydrogel.^24^, ^94^ Zhang et al. introduced tannic acid (TA) into the gelatin (Gel) hydrogel network to prepare a low swelling (25%) hydrogel GelTA.^94^ Due to the strong hydrogen bonds between TA and Gel in GelTA, the compactness of the hydrogel was increased, and the penetration of water into the hydrogel was restricted, thereby reducing the swelling of the hydrogel (Figure 7B). Liang et al. used acrylic acid (AAc), CS, and TA to prepare low‐swelling hydrogel Polymer‐AAc/CS/TA (PAAc/CS/TA).^95^ In this hydrogel, the CS chains entangled with each other and TA provided non‐covalent interactions, which increased the cohesion and reduced the swelling rate of the hydrogel. Since Cl^−^ and the N‐glucosamine on the CS chain cannot attract each other in DIW, the swelling rate of hydrogel in DIW was slightly higher than that in PBS (Figure 7C). In addition, there was no big difference in the stretchability of PAAc/CS/TA before and after swelling. Overall, the preparation of an anti‐swelling hydrogel can increase the underwater life of a hydrogel‐based device. It is also a prerequisite for the mechanical stability of the adhesive hydrogel for use in humid environments.

### 3.5 Conducive adhesive hydrogels

Adhesive hydrogels with both signal acquisition and electrical stimulation functions are one of the hotspots of current research. Significantly, the adhesive hydrogel with electronic conductivity can be obtained by introducing conductive fillers or conductive network into the hydrogel matrix, such as poly(3,4‐ethylenedioxythiophene):poly(styrene sulfonate) (PEDOT:PSS),^96^, ^97^ MXene,^50^, ^98^ reduced graphene oxide,^99^, ^100^ ions.^27^, ^101^ According to the conductive mechanism, conductive adhesive hydrogels have the distinction of ion conduction and electronic conduction. Therefore, in addition to the high conductivity, it is also necessary to consider the conductive path in a practical application. For example, adhesive hydrogels based on ion conductive paths can transport mobile ions like biological tissues. This conduction path can help construct implantable flexible electronic devices that require lower interface voltages. Indeed, conductive adhesive hydrogels have broad prospects for applications in health monitoring,^40^ human‐computer interaction,^3^ electronic skin,^102^ and stimulating or repairing nerve tissue.^103^ In particular, tissue adhesive hydrogels with high electronic conductivity have great potential in implantable and wearable bioelectronics for personalized medicine due to their intrinsic advantages such as excellent biocompatibility and tissue‐like mechanical properties.

**FIGURE 8:**
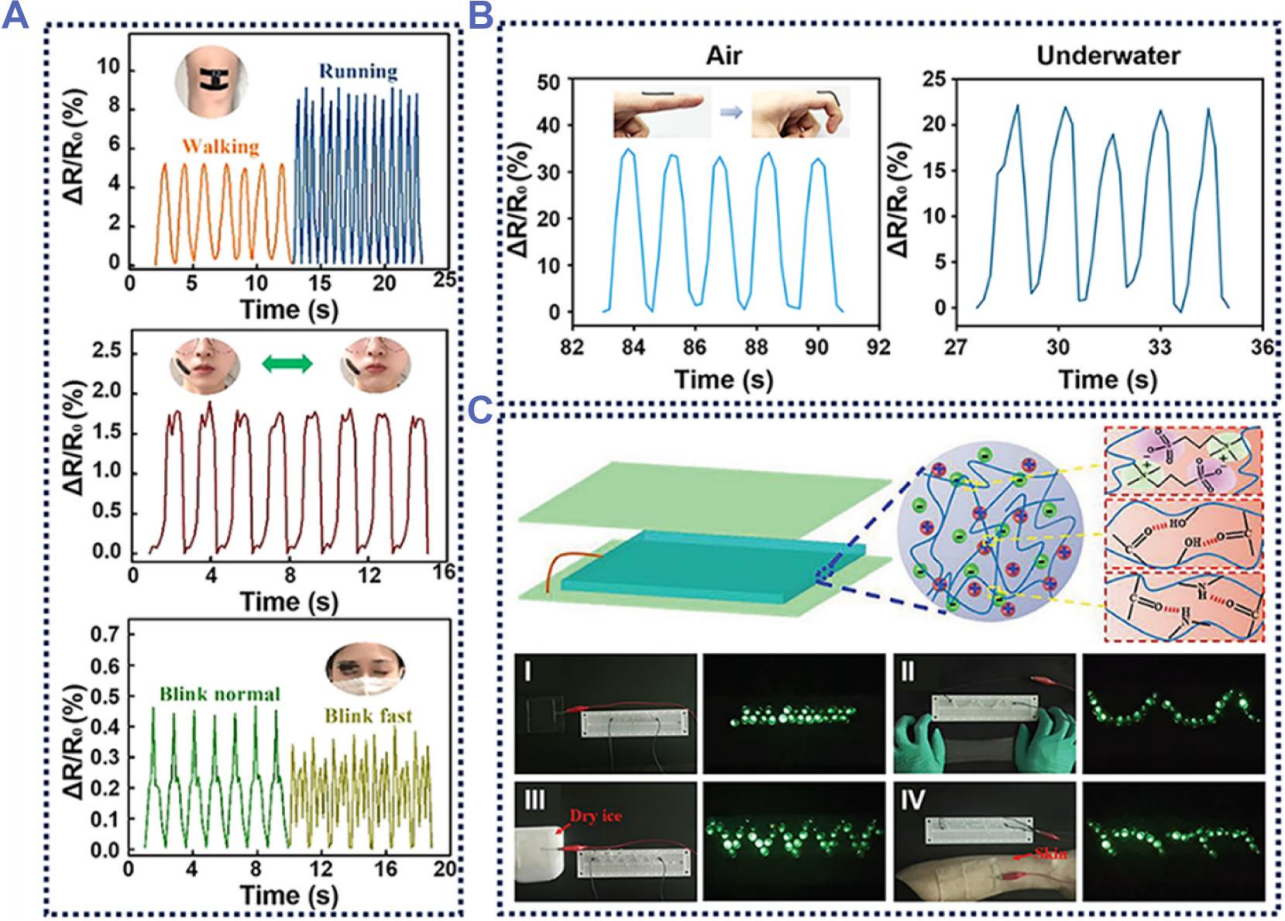
Conductive adhesive hydrogels. (A) A conductive hydrogel adhesive used for human micromotion detection, such as walking and running, inhaling and exhaling. Reproduced with permission from Ref. 104. Copyright 2022, American Chemical Society. (B) A type of hydrogel adhesive used for underwater human motion detection. Reproduced with permission from Ref. 24. Copyright 2022, American Chemical Society. (C) An AD‐TENG based on conductive hydrogel. AD‐TENG can power LED lights not only when stretched or adhered to the skin, but also at low temperatures. Reproduced with permission from Ref. 105. Copyright 2022, Wiley‐VCH. AD‐TENG, anti‐freezing degradable triboelectric nanogenerator.

Dong et al. fabricated a double‐network conductive hydrogel using graphene and PEDOT:PSS as conductive fillers.^104^ The hydrogel acted as a strain sensor that was used to detect human movement in real time. The different resistance variation values in the test results could be used to distinguish between the walking and running states of the tested human body (Figure 8A). The hydrogel‐based sensors were also able to precisely detect changes in facial expression, and even distinguish the magnitude and speed of these changes. This provided a new idea for facial expression recognition and analysis. He et al. added MXene nanosheets to the hydrogel matrix to prepare an underwater adhesive conductive hydrogel.^24^ This hydrogel was used as a stable strain sensor in both air and water environments. When the sensor was applied to the finger joint, the joint movement could be detected in both air and water conditions and could maintain a stable output (Figure 8B). It offers a new application scenario for conductive hydrogels. Guo et al. developed an anti‐freezing degradable triboelectric nanogenerator (AD‐TENG) using a zwitterionic network hydrogel.^105^ This AD‐TENG was effectively used as a practical energy source. The charged AD‐TENG could illuminate the connected LED light set in various states, including the original state, the stretched state, the low‐temperature state, and the skin‐attached state (Figure 8C). In addition to electrical conductivity and antifreeze properties, this hydrogel also had self‐healing properties and excellent mechanical properties. These properties gave this hydrogel great potential for application in human‐computer interaction devices. The main challenge of conductive adhesive hydrogel is the mismatch between high conductivity, mechanical strength, and bioactivity caused by conductive fillers.

### 3.6 Self‐healing adhesive hydrogels

In recent years, the development of self‐healing hydrogels with tunable properties that exhibit autonomous self‐healing or self‐recovery functions has attracted much attention for various applications, including soft robotics, tissue engineering, and electronic sensors. The self‐healing ability of a hydrogel is that it can intrinsically and automatically repair damage to return to its original performance under a pre‐defined set of conditions.^106^, ^107^ The self‐healing properties of the adhesive hydrogel can expand the range of applications and the duration of use. In addition, by taking advantage of the self‐healing functions, such adhesive hydrogels can improve the reliability and safety of materials in specific application scenarios by preventing failure due to crack (or fracture) accumulation. The mechanisms of self‐healing hydrogel materials are reliant on the reversible cross‐linking between adjacent molecules. Therefore, hydrogels with physical crosslinks (e.g. hydrogen bonds^108^ and van der Waals forces^109^) or dynamic chemical crosslinks (e.g. Schiff base reaction^110^ and boronic ester bonds^111^) are widely used to develop self‐healing functions.^21^

**FIGURE 9:**
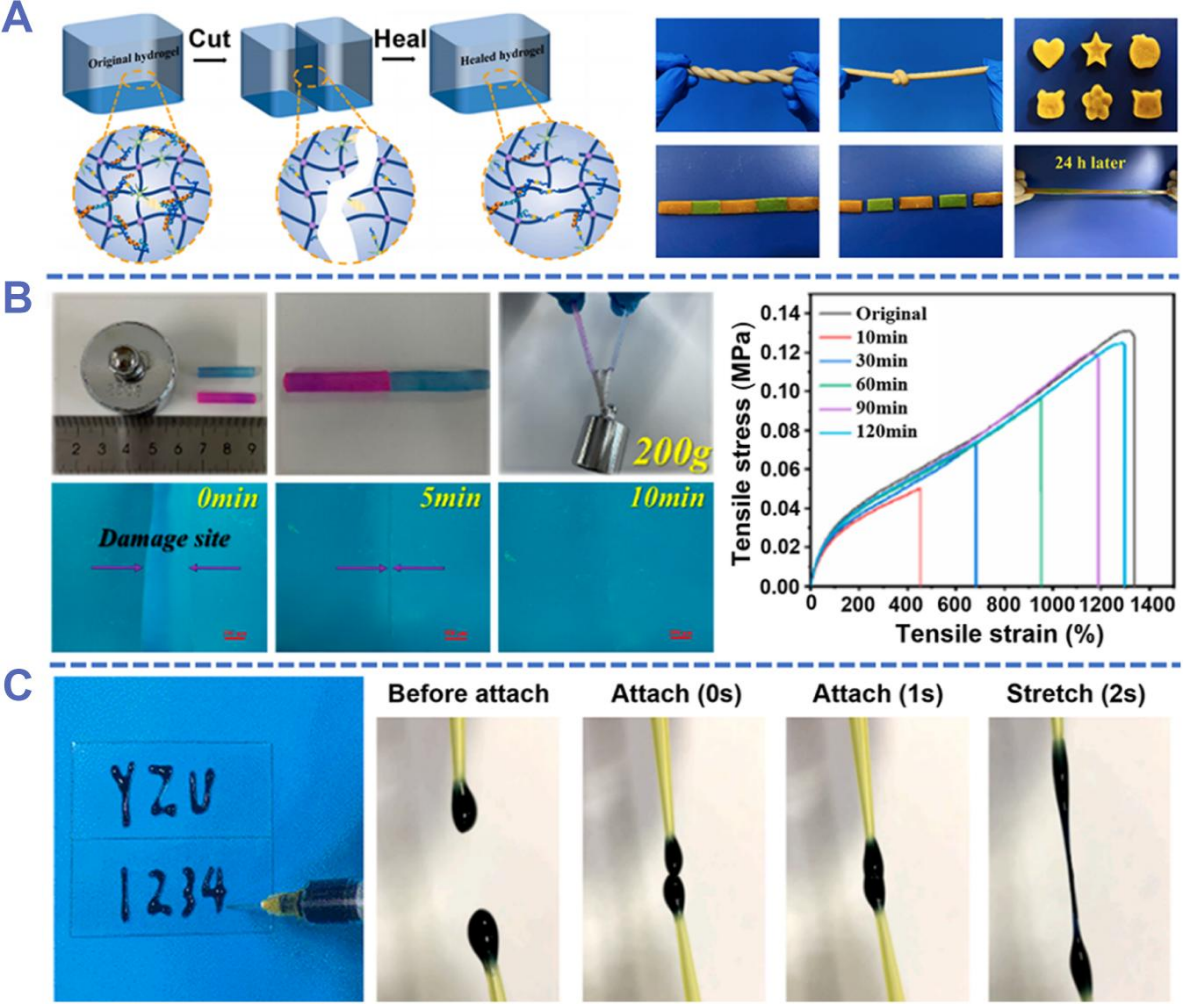
Self‐healing and injectable adhesive hydrogels. (A) A self‐healing hydrogel adhesive based on hydrogen bonds. Such hydrogen bonds from TA‐PAAm‐SPI can self‐heal within 24 h. Reproduced with permission from Ref. 112. Copyright 2022, Elsevier. (B) A self‐healing hydrogel with rapid healing capabilities. The tensile stress of this hydrogel can be almost restored within 2 h. Reproduced with permission from Ref. 113. Copyright 2022, American Chemical Society. (C) An injectable self‐healing hydrogel based on *γ*‐GM‐P. A certain degree of extensibility can be achieved within 2 s after contact with *γ*‐GM‐P. Reproduced with permission from Ref. 114. Copyright 2022, Elsevier.

Huang et al. prepared a double‐network adhesive hydrogel TA‐PAAm‐SPI using TA, AAm, and soy protein isolate (SPI).^112^ The TA‐PAAm‐SPI hydrogel not only has excellent mechanical properties, but also shows satisfactory adhesion to muscle tissue, metal, glass, and other materials. TA‐PAAm‐SPI contained a large number of hydrogen bonds, which reformed after contact, thereby promoting the self‐healing of the hydrogel. Without external stimuli, the cut hydrogels healed themselves and could be stretched after being assembled for 24 h (Figure 9A). However, TA‐PAAm‐SPI had a longer self‐healing time and a lower healing efficiency (<50%). A PVA_Borax_/AAm/SBMA‐LiCl (P_B_AS‐Li) hydrogel polymerized from polyvinyl alcohol (PVA), borax, AAm, zwitterionic polymer 3‐[N,N‐Dimethyl‐[2‐(2‐methylprop‐2‐enoyloxy)ethyl]ammonium]propane‐1‐sulfonate inner salt (SBMA), and LiCl could significantly reduce the self‐healing time.^113^ When the hydrogel was broken, the hydrogen bonds and borate bonds within the hydrogel were also broken. After a period of reassembly, these reversible interactions were re‐established. After 2 h, the hydrogel's tensile elongation had recovered to 97% and its tensile stress to 95% of its original value. The healed hydrogel can be stretched and lifted objects with a mass of 200 g (1300 times the mass of the hydrogel). Scratches on the surface of this hydrogel can be repaired to almost invisible marks within 10 min (Figure 9B). In addition, the addition of LiCl makes P_B_AS‐Li hydrogel conductive and anti‐freezing, giving P_B_AS‐Li hydrogel great application potential in flexible electronics.

On the other hand, by regulating the hydrogel's gelation conditions, composition, concentrations, and timing, injectable hydrogels can be created. The injectable adhesive hydrogels can be used as a scaffold for wound healing with different shapes, avoiding potential mismatch and discomfort to tissue caused by rigid scaffolds. Zhang et al. prepared an injectable hydrogel *γ*‐PGA/PEDOT:PSS (*γ*‐GM‐P) using *γ*‐polyglutamic acid (*γ*‐PGA) and PEDOT:PSS.^114^ The hydrogen bond interaction between *γ*‐PGA and PEDOT:PSS imparts excellent adhesion, mechanical properties, and self‐healing properties to the hydrogel. The hydrogel sample can achieve preliminary self‐healing with certain mechanical properties within 2 s, and can again conduct electricity as a conductor within 10 s (Figure 9C). In addition, the excellent stability, conductivity, and biocompatibility of *γ*‐GM‐P make it widely used in health monitoring, human‐computer interaction, and other fields.

### 3.7 Stimuli‐responsive adhesive hydrogels

**FIGURE 10:**
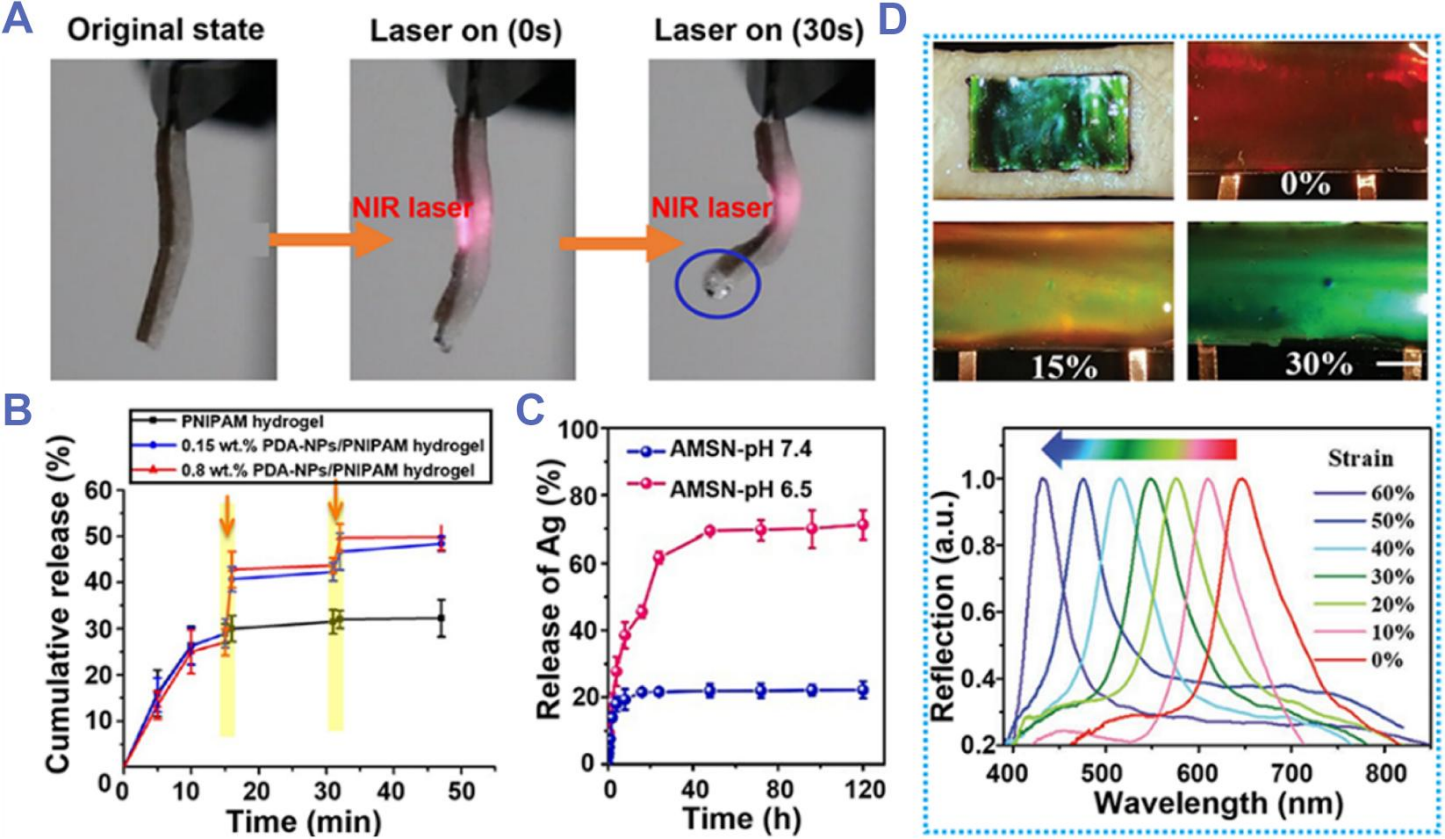
Stimuli‐responsive adhesive hydrogels. (A) A photothermally responsive hydrogel based on PDA‐NPs/PNIPAM under NIR irradiation. Reproduced with permission from Ref. 115. Copyright 2016, American Chemical Society. (B) PDA‐NPs/PNIPAM loaded with drugs can release drugs under the control of NIR. Reproduced with permission from Ref. 115. Copyright 2016, American Chemical Society. (C) A pH‐responsive hydrogel adhesive. The release of antibacterial Ag^+^ from the hydrogel can be controlled under different pH environments. Reproduced with permission from Ref. 129. Copyright 2023, Elsevier. (D) A structure color hydrogel adhesive. Such a hydrogel changes color when stretched. Reproduced with permission from Ref. 130. Copyright 2020, Wiley‐VCH. NIR, near‐infrared response.

Stimuli‐responsive hydrogels can respond to stimuli by undergoing some changes and exhibiting specific functions.^115^, ^116^ Various stimuli, including temperature response,^117^, ^118^ pH response,^119^, ^120^ magnetic response,^121^, ^122^ near‐infrared (NIR) response,^123^, ^124^ photo responsive^125^, ^126^ have been used to induce changes in one or more properties of these hydrogels. For example, NIR has a significant ability to penetrate deep into tissue, causes little damage to biological tissue, and can be applied instantly. These features make it suitable for a wide range of applications in the field of biomedicine.^115^ Han et al. prepared PDA‐NPs/PNIPAM hydrogels using polydopamine nanoparticles (PDA‐NPs) and poly(N‐isopropylacrylamide) (PNIPAM). PDA‐NPs can absorb NIR and convert the energy into heat.^127^ PNIPAM hydrogels have remarkable temperature‐triggered bulk phase transition properties.^128^ Under the NIR irradiation, the hydrogel will cause a temperature increase, resulting in volume shrinkage. When the bilayer hydrogel composed of PDA‐NPs/PNIPAM layer and PNIPAM layer was irradiated by NIR for 30 s, the hydrogel was obviously bent and water droplets could be observed (Figure 10A). The PDA‐NPs/PNIPAM hydrogel loaded with dexamethasone can only release the drug automatically for 10 min, and the drug release stops after 10 min (Figure 10B). After applying NIR to the hydrogel (yellow arrows), the hydrogel immediately started to release the drug, and when the application of NIR was stopped, the drug release stopped. Thus, drug release can be controlled by the ON‐OFF of NIR. It is worth mentioning that this hydrogel has a NIR‐assisted healing function.

In addition to controlling drug release through NIR, it can also be controlled by changing the pH of the environment. Huang et al. prepared the hydrogel PEGDA/C‐HA‐AMSN using polyethylene glycol diacrylate (PEGDA), catechol‐modified hyaluronic acid (C‐HA), and Ag‐doped mesoporous silica nanoparticles (AMSN) (Figure 10C).^129^ The AMSN framework is able to dissociate under acidic conditions, resulting in an acid‐responsive release of Ag. Under neutral conditions, the drug release time of the hydrogel could only be maintained for 20 h, and the drug release amount was approximately 20%. Under acidic conditions, 60% of the drug could be released continuously within 24 h, and the drug could be released continuously for up to 7 days. Therefore, the PEGDA/C‐HA‐AMSN hydrogel can control the delivery of antibacterial Ag according to the acidic environment of bacterial infection wounds, and promote wound healing.

It is also possible to use the response of the hydrogel to stretching as a sensor. Structural colors are the result of nanoscale periodically structured materials.^131^, ^132^ Photonic crystals display colors by reflecting specific wavelengths of visible light. If the lattice spacing is different, the colors displayed will be different.^133^, ^134^ Zhao et al. added conductive carbon nanotube polydopamine (CNTs‐PDA) to elastic polyurethane (PU) inverse opal scaffold to make the hydrogel have the dual‐signal response of real‐time color sensing and electrical signal detection (Figure 10D).^130^ The PU layer with inverse opal structure endows the hydrogel with excellent mechanical properties and bright structural color. When the hydrogel was stretched from 0% to 30%, the color of the hydrogel gradually changed from red to green. This is due to the reduction in the interplanar distance between the diffractive surfaces. Since the hydrogel contains CNTs‐PDA, stretching the hydrogel caused its color to change and its electrical resistance to change. This hydrogel has a good optical signal response and conductance response, which has great potential in flexible electronics applications.

By exploiting the response of the hydrogel, the adhesion of the hydrogel can also be controlled. Jiang et al. developed a temperature‐responsive hydrogel using gelatin methacryloyl (GelMA) chains, which dissociate at body temperature and entangle when cooled.^34^ When this adhesive hydrogel came into contact with warm skin, the adhesive properties were activated due to the binding of dissociated GelMA chains to the tissue surface. When an ice pack was placed on hydrogel, the hydrogen bonds between the entangled GelMA chains increased, making it more difficult for the hydrogel to interact with the tissue surface, resulting in painless detachment. Lee et al. exploited the ability of PNIPAM to generate volume changes with temperature changes to achieve controllable adhesion of hydrogels.^135^ This hydrogel, which can be disassembled on demand, can be painlessly disassembled without causing additional damage, providing new ideas for applications such as baby skin dressings and surgical robots.

The use of adhesive hydrogels is becoming more widespread, but different applications require hydrogels with different properties. For example, due to the high‐water content in the hydrogel, the hydrogel is easily frozen in a low‐temperature environment. Freezing hydrogels will lose their adhesion and mechanical properties, so antifreeze hydrogels have been studied extensively.^136^, ^137^ At higher temperatures, the water in the hydrogel will evaporate, resulting in reduced lifespan and even functional failure of the hydrogel. Therefore, it is very meaningful to study the high‐temperature resistance of hydrogels in order to expand the application environment of hydrogels and to ensure the stability of hydrogels.^138^, ^139^ When the transparent hydrogel is used as a wound dressing, the recovery status of the wound can be observed more clearly, which helps the user to judge the next step of treatment more quickly. Therefore, the application prospects of hydrogels with certain transparency are very broad.^101^, ^140^ Not only swelling but also excessive water loss can lead to great changes in the properties of hydrogels. Therefore, it is very important to study hydrogels with water retention.^141^, ^142^ Although many hydrogels have achieved excellent results in various properties, there is still great demand and difficulty in developing multifunctional and integrated hydrogels.^115^

## 4 APPLICATIONS OF ADHESIVE HYDROGELS IN BIO INTERFACES

Multifunctional adhesive hydrogels have a wide range of biomedical applications (Table 1). For example, conductive adhesive hydrogels have the potential to replace existing inorganic and/or metal‐based electrodes for detecting electrophysiological signals or transmitting electrical stimulation.^71^, ^105^ Wettable and adhesive hydrogels expand the application range of wearable sensors^145^ and also offer the possibility of in vivo adhesion.^151^ With respect to wound treatment, in addition to promoting the rapid healing of wounds on the skin surface,^152^, ^153^ adhesive hydrogels with excellent mechanical properties provide new options for repairing blood vessels^154^ and organ patches,^155^ and can also be used to treat corneal injuries,^51^ bone injuries,^156^, ^157^ nerve injuries.^158^ In addition, multifunctional adhesive hydrogels that can load and release drugs^159^, ^160^ and inhibit bacteria^32^ are also being researched.

**TABLE 1:** Examples of multifunctional adhesive hydrogels in biointerfaces.

Composition	Adhesive mechanism	Features	Application	Ref
THMA/PEGDA/SA	Topological entanglement, ion chelation, and hydrogen bonds	Stretchability >850%	Wound dressing	20
AAm/DA/MEA	Hydrogen bonds, coordination, cation‐π interactions, and π‐π stacking	Stretchability >900%	Motion sensor	8
ASP/AC	Hydrogen bonds	Elongation at a break of 1700%	Organ patch	67
DCMC/CS/AAc/Al^3+^	Hydrogen bonds and electrostatic interaction	Sensitivity (gauge factor [GF]) = 15.56	Motion sensor	27
*γ*‐PGA/PEDOT:PSS	Multiple hydrogen bonds	Conductivity = 12.5 S m^−1^	Triboelectric nanogenerator	114
AAc/4‐VPBA/HEMA	Hydrophobic interaction, hydrogen bonds, and chemical anchorage	Interfacial toughness of 400 J m^−2^	Electrode and in vivo blood flow monitoring	143
Gel/TA/AAc	Hydrogen bonds, hydrophobic interactions, and π‐π stacking	Fracture elongation of 2000%	Motion sensor	28
AAc/EPL/GA	Hydrogen bonds, π‐π stacking, hydrophobic interaction, and covalent bonds	Stretchability >780%	Wound dressing	4
Gel‐DA/CuPDA NPs/HA‐PBA	Schiff base, hydrogen bonds, and electrostatic interactions	Antibacterial rate >86%	Wound dressing	18
PEGDGE/CS/*γ*‐PGA	Hydrogen bonds and covalent bonds	Lap‐shear strength of 46.1 ± 1.9 KPa	Surgical adhesives	144
HACC/PAAc	Hydrogen bonds and electrostatic interaction	Tensile strength of 435 KPa	Motion sensor	145
AAc/CTAB/LMA	Dipole‐dipole interactions, electrostatic interactions, and van der Waals interactions	Adhesive strength of 644.8 ± 31.4 KPa	Underwater sensor	146
AAm/SDS/DA	Ferric coordination bond and hydrophobic interaction	Burst pressure >975 mmHg	Tissue couplant for ultrasound imaging	39
PPBA/PVA	Cation‐π interactions, π‐π stacking, and hydrogen bonds	Adhesion strength of 45 KPa	Wound dressing	147
CS/OSG	Hydrogen bonds and covalent bonds	pH‐dependent swelling property	Drug delivery system	30
PNIPAM/AAc/CS/*β*‐CD	Hydrogen bonds	Thermal contraction	Wound dressing	148
Gel/TA/borax	Schiff base, Michael addition, and hydrogen bonds	Photothermal antibacterial capacity	Wound dressing	149
AAc/DMAPS	Hydrogen bonds	Transparency >90%	Ion hydrogel triboelectric nanogenerator	136
AMPS/AAm	Microcavity topography and the interlocking effect	Temperature adaptability from −20 to 80°C	Flexible supercapacitor	150

Abbreviations: 4‐VPBA, 4‐vinylphenylboronic acid; AC, acryloyl chloride; AMPS, 2‐acrylamido‐2‐methylpropanesulfonic acid; ASP, aspartic acid; CTAB, cetyltrimethylammonium bromide; CuPDA NPs, Cu‐loaded polydopamine nanoparticles; DCMC, dialdehyde carboxymethyl cellulose; DMAPS, 3‐dimethyl(methacryloyloxyethyl)ammonium propane sulfonate; EPL, ε‐poly‐L‐lysine; GA, gallic acid; Gel‐DA, gelatin modified by dopamine; HACC, 2‐hydroxypropyltrimethyl ammonium chloride chitosan; HA‐PBA, hyaluronic acid modified by phenyl boronate acid; HEMA, hydroxyethyl methacrylate; LMA, lauryl methacrylate; MEA, 2‐methoxyethyl acrylate; OSG, oxidized succinoglycan; PEGDGE, poly(ethylene glycol) diglycidyl ether; PPBA, poly(N,N‐dimethylethylenediamine‐g‐3‐bromomethylphenylboronic acid) phosphazene; SA, sodium alginate; SDS, sodium dodecyl sulfate; THMA, N‐[Tris (hydroxymethyl) methyl] acrylamide.

### 4.1 Flexible sensors

**FIGURE 11:**
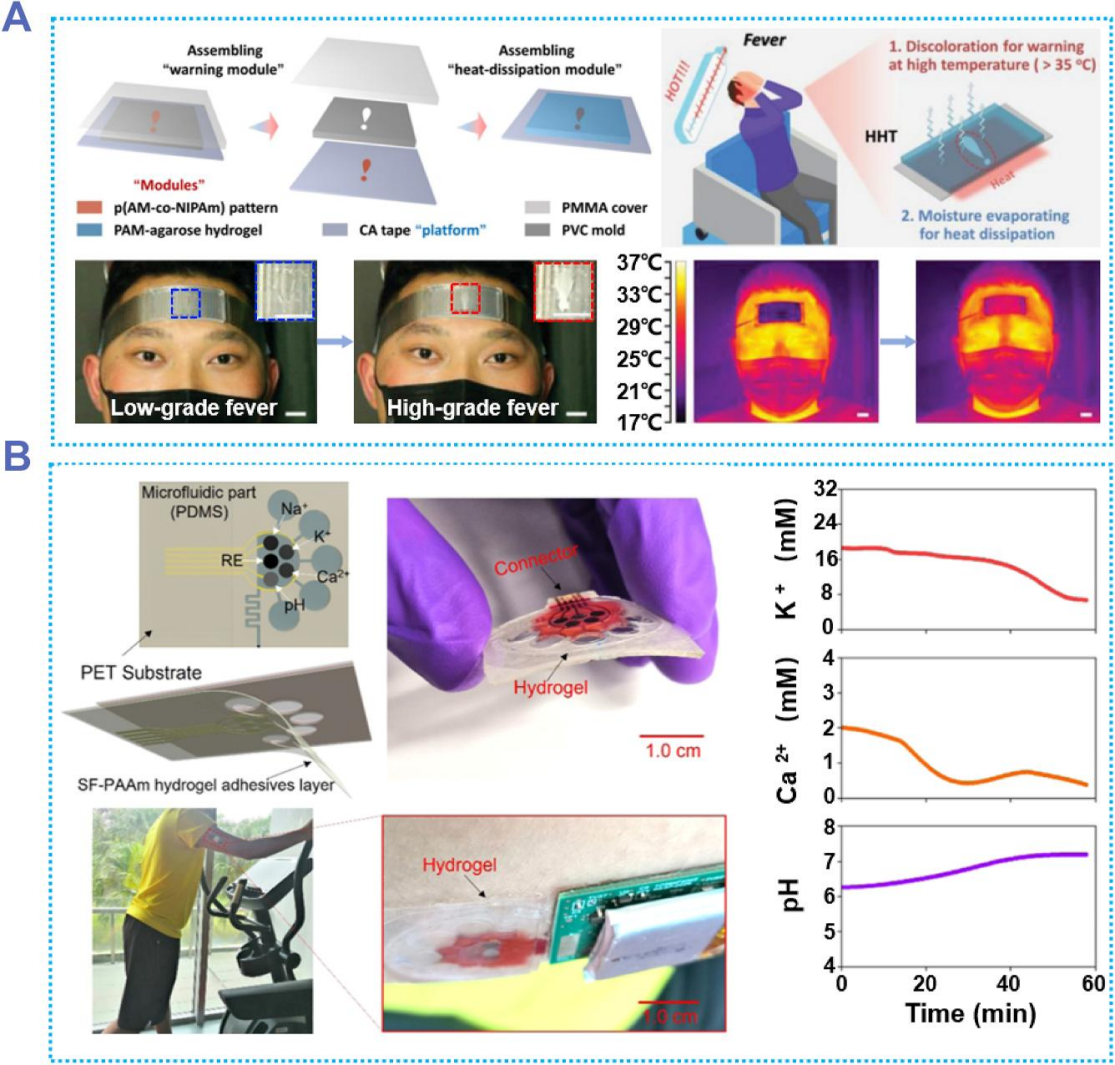
Flexible sensors based on adhesive hydrogels. (A) Adhesive hydrogel for a forehead temperature sensor. The hydrogel can respond to temperature changes, and if it detects that the forehead is hot beyond a certain level, a warning sign appears and assists in cooling. Reproduced with permission from Ref. 161. Copyright 2023, Wiley‐VCH. (B) Adhesive hydrogel for sweat analysis sensor. The sensor adheres firmly to the skin, even during sweating. Reproduced with permission from Ref. 37. Copyright 2022, American Chemical Society.

Flexible sensor is a multidisciplinary emerging research field involving many disciplines such as materials science, chemistry, physics, biology, and electronics. However, the widespread use of flexible sensors is currently constrained by a number of issues, such as high Young's modulus, poor responsiveness, and poor biocompatibility. Hydrogel flexible electronics could provide a new solution to the above issue due to the inherent advantages. As shown in Figure 11A, a heat‐dissipating hydrogel tape (HHT) was fabricated by pouring the temperature‐sensitive NIPAM polymer into the “exclamation mark” mold and then covering it with PAAm‐agarose hydrogel.^161^ The hydrogel rapidly became opaque when the temperature exceeded 35°C. This discoloration effect can be used to indicate excessive temperature (fever). In addition, as the temperature rises, the PAAm‐agarose hydrogel lost water, which accelerated the temperature drop. HHT can be used to warn of fever and reduce the head temperature in time to prevent brain damage from prolonged high temperatures. After using HHT for a period of time, the forehead temperature was 5°C lower than that without HHT. HHT has a surprising effect in detecting fever and helping to reduce fever. In addition to a wearable sensor for qualitative detection, Wang et al. developed a sweat sensor capable of real‐time quantitative detection using SF‐polyacrylamide (SF‐PAAm) hydrogel as an adhesive layer.^37^ The hydrogel SF‐PAAm can show strong adhesion even in a wet environment, which is the basis for the sweat‐proof function of the sensor. This sweat sensor is capable of real‐time measurement of sweat pH and concentration of ions (including Na^+^, K^+^, and Ca^2+^ ions). The results measured by the sensors can be transmitted to the data analysis component via Bluetooth (Figure 11B). This hydrogel has great application potential as a sensor adhesion material.

**FIGURE 12:**
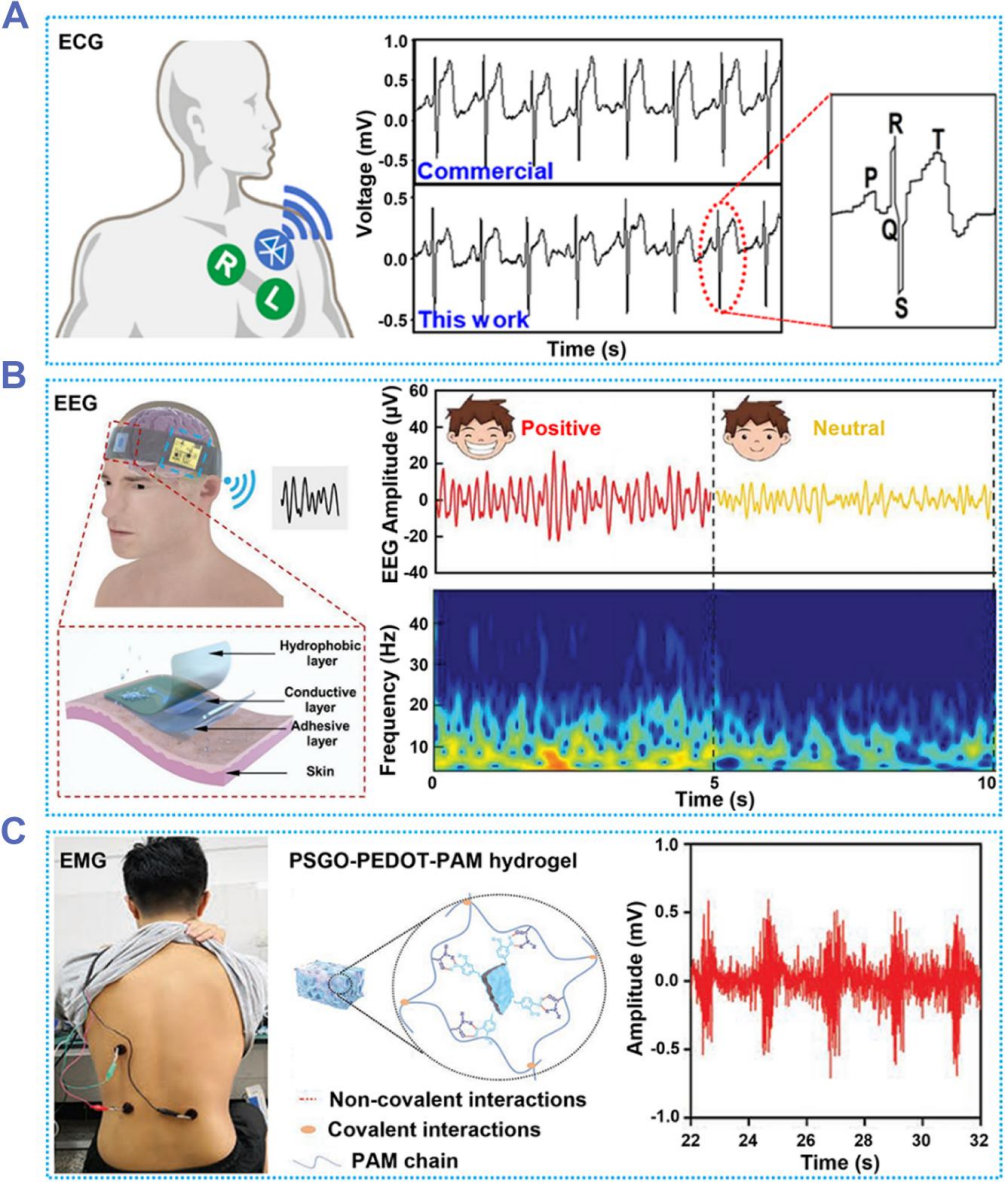
Bioelectric electrodes based on adhesive hydrogels. (A) An ECG electrode. The electrodes adhere to the skin when applied hot and are easily and residue‐free removed when applied cold. It measures an ECG comparable to that obtained with commercially available electrodes. Reproduced with permission from Ref. 162. Copyright 2022, The Authors, published by Springer Nature. (B) An EEG electrode. The simultaneous use of three electrodes can effectively improve the SNR of the electrodes. The collected EEG can be used for emotion classification. Reproduced with permission from Ref. 163. Copyright 2022, Wiley‐VCH. (C) An EMG electrode. The electrode can be firmly adhered to the skin, and the measured EMG is almost the same as that of the commercial electrode. Reproduced with permission from Ref. 164. Copyright 2020, Wiley‐VCH. ECG, electrocardiogram; EEG, electroencephalogram; EMG, electromyogram.

Accurate and timely measurement of bioelectricity is very important for daily health monitoring and clinical diagnosis. The electrophysiological signals including ECG, electroencephalogram (EEG), electrogastrogram (EGG), electromyogram (EMG), electrooculogram (EOG) signals are the important parameters of our human organism. Currently, commercially used bioelectricity measurement electrodes mainly include Ag/AgCl electrodes and stainless‐steel electrodes. However, most of these electrodes cannot move with the extrusion of the skin, cannot prevent sweat, and lack anti‐interference. This not only causes a lot of inconvenience when using the electrodes, but also results in a low SNR of the detected electrical signal. Conductive hydrogel as electrode can solve these problems well. For example, a hydrogel composed of PVA, phytic acid, and Gel (Abbreviated as PPG hydrogel) was developed to be used as an ECG signals detection electrode.^162^ When PPG was attached to the skin, it could stretch and twist with the skin. The PPG with a 3D network structure enables the electrode to exchange water vapor with the outside world and has good air permeability. It can achieve 4–55°C adhesion and can be easily removed without causing skin irritation. Overall, PPG can combine well with the skin and reduce the risk of impedance increase due to electrode separation from the skin, making it an ideal material for electrodes. Based on this PPG electrode, a portable ECG signals detection system can be made, and the detected data can be output in real time via Bluetooth (Figure 12A). The ECG signals measured by this detection system had similar accuracy and precision to the gel electrodes currently on the market. In addition, the characteristic peaks (P, Q, R, S, T waves) of the collected signals were clearly visible and corresponded to standard signals.

A device/skin interface based on an adhesive and hydrophobic bilayer hydrogel (AHBH) allows the high‐quality acquisition of electrophysiological signals.^163^ Unlike the Ag/AgCl electrode, which can become detached from the skin with perspiration, the AHBH can remain sticky in a wet environment, allowing it to remain firmly attached to the skin. Such a hydrogel can be used as a measuring electrode by combining it with electrically conductive composites. The electrical signal detected by the AHBH‐ECC electrode showed almost no difference between before and after sweat spraying, but the Ag/AgCl electrode was unable to measure the electrical signal because it dropped off after sweat spraying. Based on this electrode, a portable and wireless AHBH‐EEG detection system was proposed. This system consisted of three AHBH‐ECC electrodes, which were arranged in an orderly manner and could accurately record EEG signals without interference (Figure 12B). This system is capable of analyzing the results of the EEG signals, showing the volunteer's emotional classification (positive, neutral, negative). The accuracy rate of the system for emotion classification can reach 90%, and the high SNR signals collected by the electrodes contribute a lot to this high accuracy rate. This emotion classification system will have broad application prospects in the fields of human‐computer interaction and adjuvant therapy.

Adhesive hydrogel with high electronic conductivity can also be used to detect human motion and electrophysiological signals.^164^ The polydopamine‐reduced and sulfonated graphene oxide‐PEDOT‐PAAm (PSGO‐PEDOT‐PAAm) was used to develop a conductive adhesive hydrogel for use as an EMG electrode (Figure 12C). In this hydrogel system, PSGO‐PEDOT‐PAAm deforms as it moves with the skin, causing its electrical resistance to change, which causes a change in the electrical current in the circuit. The current‐time curve of this system could reflect the movement of the human body. In addition, this flexible hydrogel could also be used to detect the EMG signals of human muscles in a relaxed state. The quality of EMG signals measured by PSGO‐PEDOT‐PAAm electrode was almost equal to that of commercially available electrodes. Moreover, since the PSGO‐PEDOT‐PAAm has better adhesion, ductility, and biocompatibility, this electrode can have better applications in the field of electrophysiological signal detection.

### 4.2 Interface bioelectronics

**FIGURE 13:**
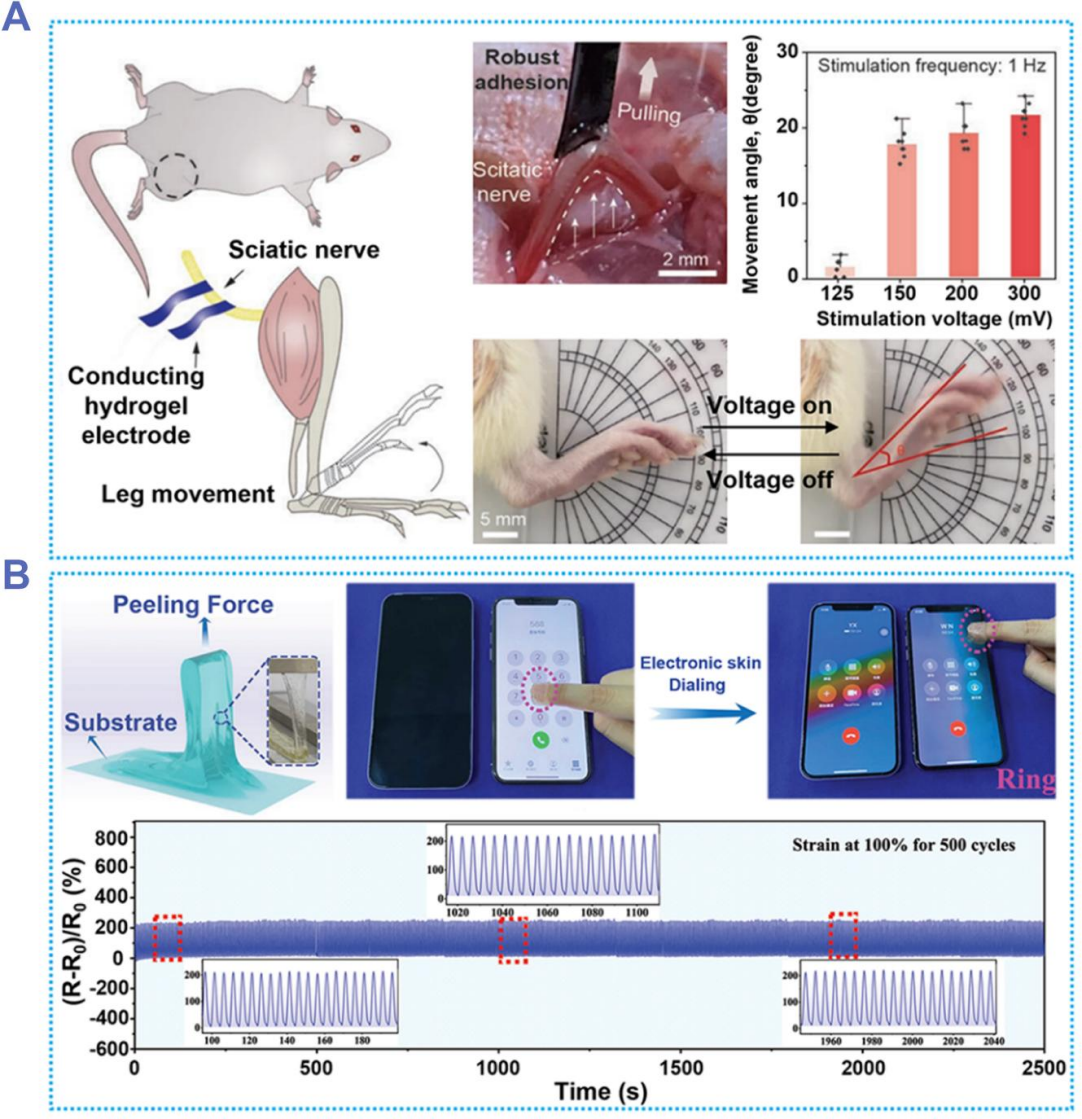
Tissue interface bioelectronics based on adhesive hydrogels. (A) Adhesive hydrogel electrode for electrical stimulation. The hydrogel can firmly adhere to the nerve. After a voltage is applied to the electrodes, the mouse's leg moved in response to the voltage. Reproduced with permission from Ref. 165. Copyright 2022, Wiley‐VCH. (B) Conductive hydrogel for electronic skin. This hydrogel could be used to operate smartphones. The hydrogel has good anti‐fatigue performance, and the electrical resistance is almost unchanged in the 500‐cycle stretching test. Reproduced with permission from Ref. 166. Copyright 2022, Wiley‐VCH.

Interface bioelectronic devices that can be in close contact with biological tissues are now widely used in applications such as diagnosis, treatment, and electrical signal acquisition due to the rapid advances in miniaturization and flexible electronics. In practical applications, it is essential to establish a conformal and stable contact between the electronic device and the target tissue to ensure the reliable performance of bioelectronic devices. Therefore, adhesive hydrogels can play an important role in the field of interface bioelectronics. Li et al. developed a PEDOT:PSS/PVA hydrogel that not only has excellent mechanical properties, but is also highly conductive, providing a new option for flexible electronics.^165^ PEDOT:PSS/PVA hydrogel can realize the function of implantation and detection of EMG signals. The EMG signals recorded on the days 7 and 14 after implantation had similar SNR, indicating that this electrode has good in vivo stability. In addition to detecting bioelectricity, medical electrodes can also be used as stimulating electrodes to apply current or voltage to organisms. PEDOT:PSS/PVA was able to adhere firmly to the surface of the sciatic nerve (Figure 13A). The voltage could apply to the nerve through the hydrogel. The mouse's leg began to move at a voltage of 125 mV, and when the voltage increased to 300 mV, the leg had moved 22° from its initial position. However, the working voltage using the currently available graphene hydrogel as a stimulation electrode is about 750 mV.^167^ This may be due to the higher conductivity of the PEDOT:PSS/PVA hydrogel. In conclusion, the PEDOT:PSS/PVA hydrogel has potential for further application in signal detection and neuromodulation.

Conductive adhesive hydrogels can be used not only as medical electrodes but also as electronic skin, which has great potential in human‐computer interaction applications. The PAAm/phenylboronic acid‐ionic liquid/cellulose nanofibrils (PAAm/PBA‐IL/CNF) hydrogel developed by Yao et al. can be used as an electronic skin to replace fingers for operating electronic devices.^166^ PAAm/PBA‐IL/CNF can maintain a large adhesive force on different substrates and can be repeatedly adhered. In addition, 100% strain was continuously applied to the hydrogel for 500 cycles, and the resistance of the hydrogel did not fluctuate significantly, indicating that the hydrogel has good stability. Adhering the hydrogel to a finger or a pen can be used to operate a mobile phone, make a phone call, and draw a picture (Figure 13B). This hydrogel will have broad prospects in the application of electronic skin and wearable devices.

### 4.3 Tissue engineering

**FIGURE 14:**
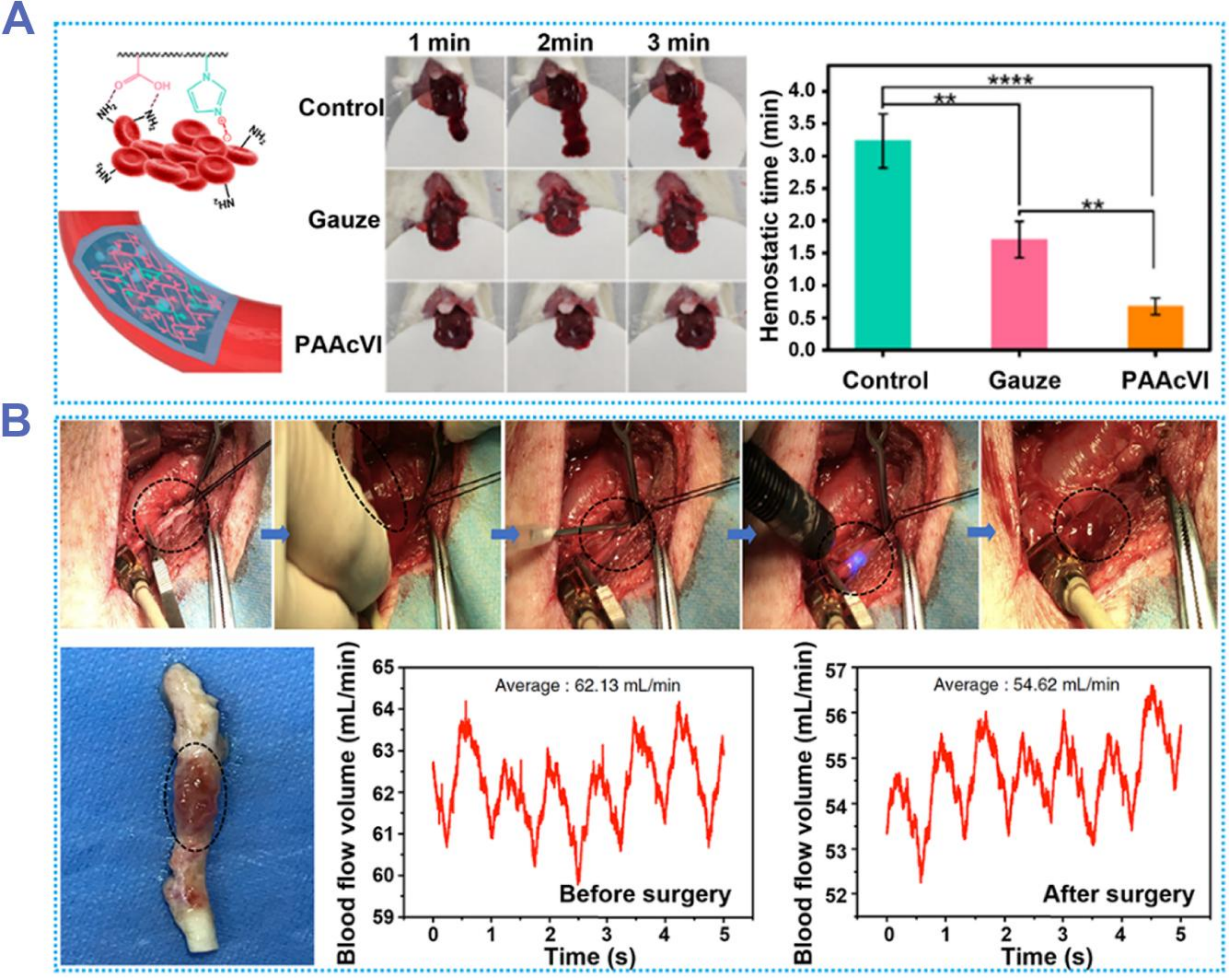
Materials for hemostasis and blood vessel repair based on adhesive hydrogels. (A) A PAAcVI hydrogel used for hemostasis. Such PAAcVI hydrogel can greatly shorten the hemostasis time of the liver. Reproduced with permission from Ref. 168. Copyright 2022, American Chemical Society. (B) An adhesive hydrogel both for hemostasis and blood vessel sealing. The hydrogel can be gelled within 1 s and adhere to blood vessels for more than 14 days, which greatly improves the efficiency of sealing blood vessels and promoting blood vessel healing. Reproduced with permission from Ref. 169. Copyright 2019, The Authors, published by Springer Nature. PAAcVI, poly(acrylic acid‐co‐1‐vinylimidazole).

In addition to use in hydrogel electronics, adhesive hydrogels can be used in bioengineering applications such as hemostasis and repair of blood vessels or organs. Wang et al. developed a poly(acrylic acid‐co‐1‐vinylimidazole) (PAAcVI) hydrogel that can be used to stop bleeding.^168^ PAAcVI hydrogel has excellent mechanical properties and strong wet adhesion. It is worth mentioning that the adhesion and disaggregation of PAAcVI can be controlled by Zn^2+^, because the imidazole group contained in the hydrogel is very sensitive to Zn^2+^. The hemostatic ability of the hydrogel can be evaluated using a mouse liver hemorrhage model. In the mouse model, the control group had obvious bleeding phenomenon. The gauze‐assisted hemostasis group achieved hemostasis in about 1.5 min, and the bleeding volume was about 300 mg. In the PAAcVI group, hemostasis was achieved within 1 min (Figure 14A). The hemostatic function of PAAcVI may be due to physical adhesion to blood vessels and binding to hemoglobin.

Hemostatic adhesive hydrogel can repair blood vessels, heart, and other organs. Inspired by the ECM, Hong et al. developed a light‐responsive adhesive hydrogel for sealing hemostasis (Figure 14B).^169^ It can gel rapidly under UV light in less than 1 s and seal the wall of the artery. After the porcine carotid artery was punctured, the hydrogel was injected into the wound and irradiated with ultraviolet rays for 3–5 s, and there was no blood leakage after 30 s. The blood volume flow rate decreased after surgery, but the integrity of the blood flow system was maintained. After 2 weeks of repairing blood vessels with hydrogel, it was observed that the hydrogel still adhered to the damaged blood vessel, and the wound began to heal. This hydrogel will have great application potential in the treatment of human blood vessels and heart.

**FIGURE 15:**
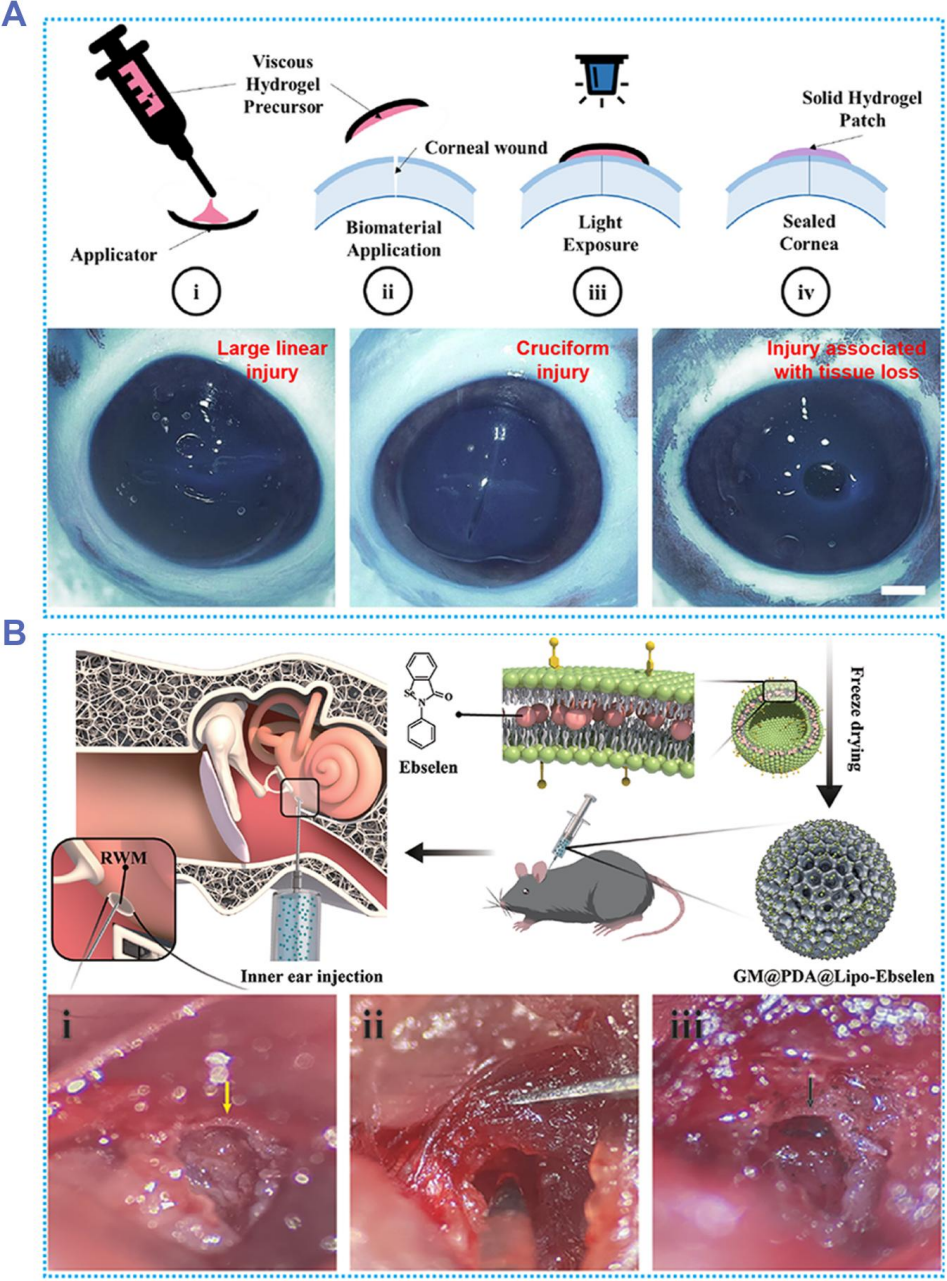
Adhesive hydrogels for organ repair and drug delivery. (A) A hydrogel capable of sealing corneal wounds, including linear injury, cruciform injury, and circular injury. Reproduced with permission from Ref. 171. Copyright 2022, Elsevier. (B) A hydrogel capable of loading and releasing drugs. Injection of the hydrogel at the round window membrane can effectively treat noise‐induced hearing loss. Reproduced with permission from Ref. 172. Copyright 2022, Wiley‐VCH.

In addition to repairing blood vessels and organs, adhesive hydrogels can be used to repair oral wounds and the corneas.^92^ Existing treatments can seal small corneal lesions, but repairing large lesions is difficult.^170^ Jumelle et al. developed an adhesive hydrogel that can seal complex open eye injuries.^171^ This hydrogel can firmly adhere to the surface of the eyeball, and has sufficient mechanical properties to withstand eyeball pressure. The hydrogel precursor was applied to the eyeball injury using an applicator to assist application, and the hydrogel could successfully seal the injury after 4 min of light exposure (Figure 15A). In vitro, it could block linear injury, cruciform injury, and injury associated with tissue loss. It was able to cover the entire wound and fit close to the edges without leakage. The hydrogel effectively sealed open lesions in the eye. However, it did not have high transparency, which resulted in reduced patient vision during the wound healing phase. The transparency of corneal repair hydrogels remains an important research direction.

Adhesive hydrogels capable of drug release are also widely used in biomedical fields. The noise‐induced hearing loss (NIHL) increases with the noise exposure levels.^172^ The drug Ebselen has been shown to prevent NIHL in clinical trials.^173^ Chen et al. grafted PDA onto the surface of methacrylonitrile acylation gelatin microspheres (GM) and loaded drug ebselen liposomes (Lipo‐ebselen) to develop a drug‐releasing hydrogel GM@PDA@Lipo‐ebselen.^172^ The GM@PDA hydrogel could firmly adhere to the round window membrane (RWM), which provided conditions for the hydrogel to release drugs at a fixed point and prolonged the release time. GM@PDA@Lipo‐ebselen was injected into the RWM of the inner ear of mice with NIHL (Figure 15B). Compared with the sham group, GM@PDA‐injected group, PBS‐injected group, and ebselen‐injected group, the hearing of mice injected with GM@PDA@Lipo‐ebselen was significantly improved on the third day of injection. On the 14th day of injection, the hearing was not significantly different from the sham group. The drug‐loaded adhesive hydrogel significantly improved hearing in mice with NIHL. This hydrogel has great potential for clinical application.

## 5 CONCLUSION AND PERSPECTIVE

Adhesive hydrogel, one of the most important building blocks of interface materials, will be a bright spot for their continuous and steady progress and pave the way for its practical application in the fields of tissue scaffolds, wound healing, soft robots, and chemical/physical sensors.^7^, ^52^, ^174^, ^175^ By combining with chemical bonding, physical interactions, and bioinspired strategies, those adhesive hydrogels with various functions will satisfy the fundamental requirements in practical applications. For example, stopping the bleeding and shielding wounds from gastric acid invasion are two of the most important procedures during endoscopic submucosal dissection. Those adhesive hydrogels featured with a rapid gel process, good bioactivity, and degradability, anti‐swelling capabilities, as well as excellent physiological stability are the candidate interface materials for intestinal repair engineering. To avoid the biocompatibility issue facing some synthetic polymers, recent research has focused on adhesive hydrogels based on natural polymers (e.g., collagen, silk fibrin, and gelatin), which aim to accelerate the pace of clinical application.^58^ By combining the SF polymer network and internal crystallization of hydrophobic moieties, Kim et al. have developed an adhesive hydrogel with regional network mutual restriction, endowing the SF‐based adhesives with excellent anti‐swelling properties.^175^ These demonstrate the adaptability of adhesive hydrogel in terms of molecular design and functional matching to the actual application functions and qualities, which is the key distinction between other inorganic materials.

It is significant to highlight that a number of hydrogel adhesives are currently commercially available (e.g., DuraSeal, Baxter International, and Integra LifeSciences). This demonstrates that the tremendous efforts of scientists have paid off, and adhesive hydrogels have practical uses in daily life. However, adhesive hydrogels still face a number of difficulties and limitations that prevent them from reaching their full practical application. The adhesive hydrogels based on chemical bonding often face obstacles in poor biodegradability of the chemical crosslinks, biotoxicity of the chemical reaction process, and the production of byproducts. Compared with chemical bonding, hydrogel adhesives with physical interactions are significantly weaker in terms of penetrating surface bound water layers, programming underwater curing and being cohesive against water corruption, which restricts the use of such kind adhesives in a wet surface or tissue interface. In terms of nature‐inspired hydrogel adhesives, one of the remaining challenges is to correlate the adhesive properties with practical application scenarios. This requires broadening the theoretical understanding of nature‐inspired adhesives beyond modeling from an engineering perspective. Additionally, it is still another challenge to integrate smart hydrogel adhesive with many features, such as tunable, reversible, and underwater adhesion, as well as the high biocompatibility and degradable properties without sacrificing other performance.

In conclusion, the fundamental requirements of adhesive hydrogels for their biological interface application include but are not limited to good biocompatible, good mechanical robustness, underwater adhesiveness, anti‐swelling, high conductivity, and other functionalities. In this review, we highlight recent advances and challenges in the design and application of adhesive hydrogels based on chemical, physical and nature‐inspired strategies, with an emphasis on the realization of the superposition of multiple practical functionalities in a single hydrogel. Overall, apart from material issues, such as physical, chemical, and physiological properties, as well as the processing procedures and preparation methods, we shall also gain a deeper understanding of biological interactions, which is critical for the design of adhesive hydrogels. We believe that adhesive hydrogel with tailored properties will be able to meet various application demands of biological interfaces.

## AUTHOR CONTRIBUTIONS

The manuscript was written through the contributions of all authors. All authors have given approval to the final version of the manuscript.

## CONFLICT OF INTEREST STATEMENT

The authors declare that there are no competing interests.
